# Ultrasensitive Quantum Sensors Based on High-Order Exceptional Bound States

**DOI:** 10.34133/research.1091

**Published:** 2026-02-05

**Authors:** Shaohui Liu, Tian Chen, Deyuan Zou, Xiangdong Zhang

**Affiliations:** Key Laboratory of Advanced Optoelectronic Quantum Architecture and Measurements of Ministry of Education, School of Physics, Beijing Institute of Technology, Beijing 100081, China.

## Abstract

High-precision sensors are of fundamental importance in modern society and technology. Although there are various schemes for the construction of sensors relying on different physical mechanisms, obtaining sensors with higher levels of sensitivity and stronger robustness has always been expected. In particular, non-Hermitian quantum sensors have recently attracted substantial attention due to their unique properties. So far, 2 types of non-Hermitian sensors based on exceptional points and topological zero modes have been realized. Here, high-order exceptional bound states with robust properties are constructed for the first time. Based on these states, we propose theoretically and demonstrate experimentally another new type of non-Hermitian quantum sensors. Such sensors not only are robust against disorders but also have unprecedented sensitivity. Their sensing performance can display the improvement of many orders of magnitude over the previous non-Hermitian sensors. Furthermore, we design and fabricate such sensors based on circuit networks. Taking weak magnetic field detection as an example, we also experimentally demonstrate their sensing capabilities. Our work opens up new avenues for the development of highly sensitive sensors, which have a wide range of applications in various fields.

## Introduction

Physical properties in many real systems, such as the presence of loss and/or gain, should be described by non-Hermitian Hamiltonians [1]. Effective non-Hermitian Hamiltonians can induce novel topological phases and unique phenomena [2–7], which do not exist in the Hermitian system. For example, the exceptional points (EPs) in non-Hermitian systems can exhibit special spectral degeneracies, at which 2 or more eigenvalues are degenerated and the related eigenvectors coalesce. The rich physics at and around EPs has been elucidated in recent years, especially the various designs of sensors related to the EP [8–35]. While the EP sensors have shown remarkable potential, their practical implementation requires precise tuning to operate at the EP, and they face the inherent challenge of noise amplification. In parallel, non-Hermitian topological states provide a fundamentally different and more robust framework for non-Hermitian sensing.

The non-Hermitian skin effect (NHSE), namely, that the majority of eigenstates of a non-Hermitian operator are localized at boundaries, has also been discovered as another manifestation of the non-Hermitian topology. Broader implications of NHSE have been under investigation [36–48], and the effect has been observed in experiments [11,49–54]. Recent studies have shown that the energy shift of a midgap non-Hermitian topological state in response to boundary perturbations can be exponentially amplified with increasing system size [55]. This effect has been experimentally demonstrated using topolectrical circuits to detect minor variations in electrical parameters [53,56,57]. Although non-Hermitian topological sensors demonstrate promising application prospects, their detectable areas are rather localized, which are limited to the edges of systems in common [53–57].

Recently, a new class of robust states known as exceptional bound (EB) states, which are distinct from the well-known topological and non-Hermitian skin boundary states, has been found [58–60]. However, these revealed that states only correspond to the case with the second-order EPs. The question is whether there exist higher-order EB states corresponding to high-order EPs, which remains unknown. If the higher-order EB states exist, would they be helpful for achieving ultrasensitive sensing?

In this work, we first demonstrate that the high-order EB states, a new class of robust quantum states, can be constructed in exceptional systems. Compared to the second-order case, high-order EB states exhibit more robust against disorders. Especially, the high-order EB states with flat spatial distributions can be realized. Based on such states, we construct a novel class of quantum sensors to achieve ultrasensitive sensing across the entire surface of the system. Their sensing performances can display the improvement of many orders of magnitude when comparing with the previous non-Hermitian sensors. Furthermore, we design and fabricate corresponding circuit network and integrated circuit sensors to experimentally observe these phenomena. Taking weak magnetic field detection as an example, we experimentally demonstrate the sensing capabilities of such circuit sensors. Thus, our work opens up the exciting possibility for realizing robust sensors with unprecedented sensitivity.

## Results

### Theory of higher-order EB states

Consider a generic Nth-order non-Hermitian Hamiltonian H, this system exhibits simultaneous coalescence of both eigenvalues and eigenvectors, characteristic of the Nth-order EP. The corresponding biorthogonal basis is constructed from the left and right eigenvectors of H, $H|\psi_{i}^{R}\rangle =\epsilon_{i}|\psi_{i}^{R}\rangle$ and $H^{†}|\psi_{i}^{L}\rangle =\epsilon_{i}^{*}|\psi_{i}^{L}\rangle$, where $\epsilon_{i}$ denotes the eigenvalue of H. These eigenvectors satisfy the biorthogonality condition $\langle \psi_{i}^{L}|\psi_{j}^{R}\rangle =\delta_{\mathrm{ij}}$. Starting from these left and right eigenvectors of the second-order Hamiltonian, EB states embodying the nonzero entanglement entropy have been reported [58–60]. Those EB states are special boundary modes arising from the asymmetric truncation of long-range correlations and defectiveness near an EP. Their corresponding truncated projector exhibits anomalous eigenvalues beyond the usual bounds of 0 and 1. Here, we provide the construction of EB states for any *N*th-order Hamiltonian as

$$P\left(k\right)=\frac{1}{2}\left(I+\frac{H}{\epsilon_{1}}\right)=|\psi_{1}^{R}\left(k\right)\rangle \langle \psi_{1}^{L}\left(k\right)|+\sum_{i\neq 1}^{N}\frac{1}{2}\left(1+\frac{\epsilon_{i}}{\epsilon_{1}}\right)|\psi_{i}^{R}\left(k\right)\rangle \langle \psi_{i}^{L}\left(k\right)|$$ (1)

where the first term in the right part is the same as the construction of EB states in the second-order Hamiltonian, and the biorthogonal projection operator for the high-order EP system is introduced by weighting $\frac{1}{2}\left(1+\frac{\epsilon_{i}}{\epsilon_{1}}\right)$ with its corresponding eigenvalues.

The Hamiltonian $\begin{array}{c} P \end{array}$ can be obtained through Fourier transform,

$$P_{\left(l_{1},\;\alpha \right)\left(l_{2},\;\beta \right)}=\sum_{k^{\prime }}P_{\alpha \beta }\left(k\right)e^{-ik^{\prime }\cdot \left(l_{1}-l_{2}\right)},$$ (2)

where the element refers to the coupling from the sublattice α of unit cell $l_{1}$ to the sublattice β of unit cell $l_{2}$. The value of α (β) is chosen within $\left[1,\;N\right]$ for the *N*-order EB system. In addition, to avoid the singularity caused by the degenerate at the EP, we introduce a small offset ∆ in the momentum space as $\;k^{\prime }=k+∆$ in the Fourier transform.

**Fig. 1.**
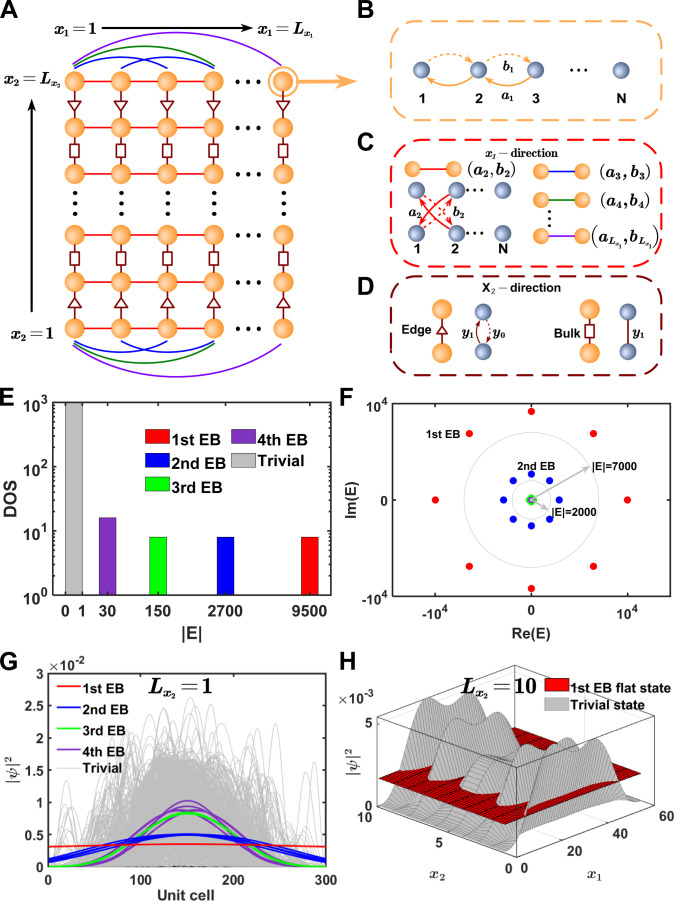
The construction of EB system. (A) Structural schematic diagram of the 2D EB system where the orange sphere represents the unit cell. The intracell couplings are shown in (B). The red, blue, green, and purple solid lines in (A) represent the NN, second NN, and third NN nonreciprocal coupling structures along the $x_{1}$ direction, whose details are shown in (C). The brown lines with triangles or rectangles represent coupling structures along the $x_{2}$ direction. The coupling details are shown in (D). (E). The EB system is one-dimensional (1D), and the corresponding density of state (DOS) is shown. Parameters are $L_{x_{1}}=300,L_{x_{2}}=1,∆=0.001,$ and N=5. (F) Eigenvalues of the 1D EB system. (G) Distributions of eigenstates for 1D EB system. (H) Distributions of eigenstates for 2D EB system. Parameters are $L_{x_{1}}=60$, $L_{x_{2}}=10$, N=5, and ∆=0.001.

In Fig. 1A, a 2-dimensional (2D) real space is considered. The scale of system is expressed as $L=L_{x_{1}}\times L_{x_{2}}$, where $L_{x_{1}}$ ($L_{x_{2}}$) is the scale along the $x_{1}\left(x_{2}\right)$ direction. The 2D momentum space is spanned by $k=\left(k_{x_{1}},\;k_{x_{2}}\right)$, which is related to $x=\left(x_{1},\;x_{2}\right)$ in 2D real space by the Fourier transform. Here, $l_{1}=\left[x_{1}\left(l_{1}\right),\;x_{2}\left(l_{1}\right)\right]$ refers to the unit cell, and $x_{1}\left(l_{1}\right)$ $\left[x_{2}\left(l_{1}\right)\right]$ represents the real space coordinate $x_{1}$ $\left(x_{2}\right)$ of $l_{1}$. The quantity $l_{2}$ is the same as above. The orange balls in Fig. 1A represent unit cells, and each cell consists of N sublattice sites that are represented by gray balls as shown in Fig. 1B. The intracell couplings ($a_{1}$ and $b_{1}$) between those adjacent sublattices are nonreciprocal, which correspond to $P_{\left(l_{1},\;\alpha \right)\left(l_{1},\;\alpha +1\right)}=a_{1}$ and $P_{\left(l_{1},\;\alpha \right)\left(l_{1},\;\alpha -1\right)}=b_{1}$, respectively.

Figure 1C shows couplings along the $x_{1}$ direction. The red solid line represents the nearest-neighbor (NN) intercell couplings that the sublattice sites are coupled with staggered sites by nonreciprocal couplings ($a_{2}$ and $b_{2}$). Here, $P_{\left(l_{1},\;\alpha \right)\left(l_{2},\;\beta \right)}=a_{2}$ and $P_{\left(l_{2},\;\beta \right)\left(l_{1},\;\alpha \right)}=b_{2}$ with $x_{1}\left(l_{1}\right)+1=x_{1}\left(l_{2}\right)$ and $x_{2}\left(l_{1}\right)=x_{2}\left(l_{2}\right)$ in $l_{1}$ and $l_{2}$. Similarly, other long-range couplings can be obtained by the similar rules. The nonreciprocal couplings are $a_{l}=\frac{1}{L_{x_{1}}}\sum_{k_{x_{1}}^{\prime }}{\left[f\left(k_{x_{1}}\;\right)\right]}^{-1/2}e^{ik_{x_{1}}^{\prime }\left(l-1\right)}\;$ and $b_{l}=\frac{1}{L_{x_{1}}}\sum_{k_{x_{1}}^{\prime }}{\left[f\left(k_{x_{1}}\;\right)\right]}^{1/2}$$e^{ik_{x_{1}}^{\prime }\left(l-1\right)}$. The term $f\left(k_{x_{1}}\;\right)={\left[2\left(1-\cos\;k_{x_{1}}\;\right)\right]}^{B}$ is taken, and B represents the mode when the system gradually reaches EP. The selection of $f\left(k_{x_{1}}\;\right)$ is that it possesses the most local Fourier coefficients [54]. Here, B=4 is taken, which represents the minimal exponent ensuring maximally identical coefficients $a_{l}$. In such a case, all of the $a_{l}$ are assigned identical values that $a_{1}=a_{2}=\ldots =a_{L_{x_{1}}}=a=\frac{1}{2L_{x_{1}}}{∆}^{-4}$ when considering ∆ is a small offset, and for the couplings $b_{l}$, $\begin{array}{c} b_{0}=3,b_{1}=-2,b_{2}=\frac{1}{2}, \end{array}$$\begin{array}{c} b_{3}=\ldots =b_{L_{x_{1}}}\;\approx 0 \end{array}$. In Supplementary Information S1, we show the different forms of $f\left(k_{x_{1}}\right)$ and related coupling coefficients.

In Fig. 1D, the NN intercell couplings in the $x_{2}$ direction are shown**.** The couplings are nonreciprocal $\left(y_{0}\;\text{and}\;y_{1}\right)$ at the edge of the *x*_2_ direction, $\begin{array}{c} P_{\left(l_{1},\;\alpha \right)\left(l_{2},\;\alpha \right)}=y_{0}, \end{array}$ direction that which satisfies $\begin{array}{c} x_{2}\left(l_{1}\right)=1\;\text{and}\;x_{2}\left(l_{2}\right)=2,\text{or}\;x_{2}\left(l_{1}\right)=L_{x_{2}}\;\text{and}\;x_{2}\left(l_{2}\right)=L_{x_{2}}-1. \end{array}$ The reciprocal couplingsare trivial at the bulk of the *x*_2_ $\begin{array}{c} P_{\left(l_{1},\;\alpha_{i}\right)\left(l_{2},\;\alpha_{i}\right)}=y_{1}\;\text{where}\;x_{1}\left(l_{1}\right)=x_{1}\left(l_{2}\right)\;\text{and}\;x_{2}\left(l_{1}\right)+1=x_{2}\left(l_{2}\right), \end{array}$$\begin{array}{c} \text{or}\;x_{2}\left(l_{1}\right)-1=x_{2}\left(l_{2}\right)\;\left(2\leq x_{2}\left(l_{1}\right)\leq L_{x_{2}}-1\right). \end{array}$ We engineer nonreciprocal couplings at the edge of x_2_direction to achieve a uniform coupling structure in real space along that direction. This prevents any undesired edge-induced modulation, thereby flattening the spatial distribution of the EB states and ensuring the flat spatial distribution across the entire 2D plane.

After the above construction, we further explore the properties of *P*. Setting $\begin{array}{c} L_{x_{2}}=1 \end{array}$yields a 1D *N*th−order EB system (*x*_1_ direction chain in Fig. 1A). Numerical diagonalization of P gives eigenvalues and eigenstates for arbitrary N (details in Supplementary Information S2). Figure 1E shows the density of states (DOS) for the 1D fifth-order EB system with $L_{x_{1}}=300$ and ∆=0.001. Besides the trivial states confined within [0, 1], 4 isolated EB eigenvalues emerge far outside this range, marked by red (first), blue (second), green (third), and purple (fourth). Each kind corresponds to multiple eigenvalues and eigenstates as shown in Fig. 1F and G. In Fig. 1F, colored dots (e.g., 8 dots for the first order) match the DOS peaks in Fig. 1E. Notably, higher-order EB eigenvalues $\left(\left|E\right|\right)$ exhibit larger separations, with the first-order EB states being the most isolated—enhancing their robustness, unlike lower-order states. Figure 1G displays the whole state distributions of the EB system with respect to unit cell index. Remarkably, the first EB state exhibits a well-defined flat spatial distribution.

For $L_{x_{2}}\neq 1$, the system becomes 2D EB system with eigenvalues and eigenstates obtained by numerical diagonalization (details in Supplementary Information S2). As shown in Fig. 1H with $L_{x_{1}}=60,L_{x_{2}}=10,$ and ∆=0.001, the 2D system exhibits the characteristic similar to the 1D case, with the first EB states showing flat spatial profiles (red) versus the heavily fluctuating trivial states (gray).

Building upon these 1D and 2D results, we emphasize that our construction method can generalize to arbitrary dimensions. Using eigenvalues and eigenstates of *N*th−order EP system $H_{0}$, the approach remains valid via the Fourier transform in higher-dimensional spaces (Supplementary Information S3).

### Robust properties of higher-order EB states

**Fig. 2.**
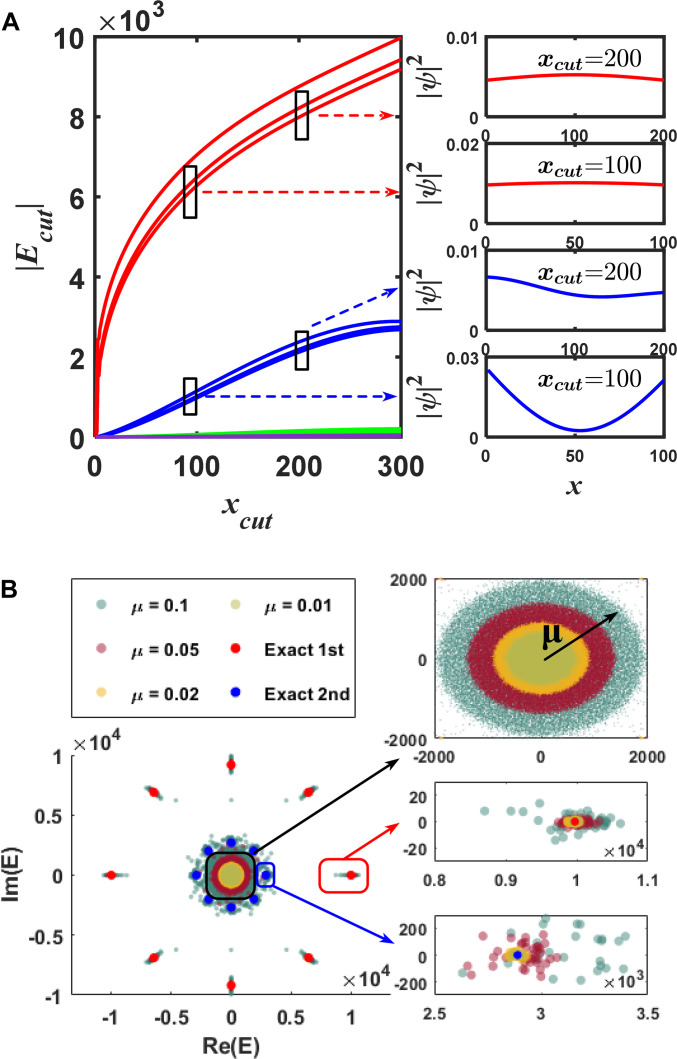
Robustness of EB states. (A) Spectral flow of the absolute value of eigenvalues $\left|E_{\mathrm{cut}}\right|$ for our system as the untruncated region $\left[x_{L},\;x_{R}\right]=\left[1,\;x_{\mathrm{cut}}\right]$ is varied. The eigenstates of different EB states are also shown when $x_{\mathrm{cut}}$ takes different values. (B) Distribution of the E eigenvalues from 40 instances of different random disorders with $L_{x_{1}}=300$.

We demonstrate the robustness of higher-order EB states following the EB spectral flow under spatial truncation [41]. The real-space cut can lead to a new non-Hermitian $P_{\mathrm{cut}}=RPR$, where R indicates the cut operation. Figure 2A shows the calculated results for the 1D EB system $P_{\mathrm{cut}}$, where the absolute eigenvalues $\left|E_{\mathrm{cut}}\right|$ of $P_{\mathrm{cut}}$ evolve as the truncation cut $x_{\mathrm{cut}}$ varying from 2 to 300 (we fix the untruncated region $\left[x_{L},\;x_{R}\right]=\left[1,\;x_{\mathrm{cut}}\right]$). This EB spectral flow is reminiscent of the spectral flow of topological states as flux is threaded. The existence of this spectral flow implies that the EB state that is well-defined at a large truncated system position $x_{\mathrm{cut}}$ is adiabatically connected to a qualitatively similar EB state at a very small truncated system with $x_{\mathrm{cut}}\propto O\left(1\right)$ when the size of lattice approximates a continuum. Three red curves in Fig. 2A means the change of 8 eigenvalues of the first EB states with the cut position. Even if there are 3 different absolute values for these first EB states, they are all distributed flat on the spatial positions. In the right part of Fig. 2A, the spatial lattice profiles of the first EB states are shown at $x_{\mathrm{cut}}=100$ and 200, respectively. When comparing these 2 distributions, it is found that the same flat spatial profile is maintained, which means that the first EB states have good robustness against spatial truncation.

The robustness of these EB states can be further demonstrated by analyzing the response of system against hopping disorder. We introduce disorder by multiplying all coupling terms by a random factor $1+r_{R}$, where $r_{R}$ is a random variable uniformly distributed over the interval $\left[-\mu ,\;\mu \right]$. In this way, the element $P_{\mathrm{ij}}$ changes to $P_{\mathrm{ij}}\left(1+r_{R}\right)$. The range of disorder μ is chosen to be μ=0.01,0.02,0.05, and 0.1. As shown in Fig. 2B, at zero disorder, the eigenvalues E of the first and second EB states are plotted by red and blue dots. The details about the third and fourth EB states and the bulk states are indicated in the black box (top right of Fig. 2B**)**. It is shown that with the increase of μ, the fluctuation near the initial eigenvalue also increases. However, it is obvious that the eigenvalues of the first and second EB states are separated from the other eigenvalues, and the first EB state is further separated. The corresponding details of the first and second EB states are shown in the middle right and bottom right of Fig. 2B. It can be seen that the first and second EB states are still robust even though the largest disorder exists (μ=0.1).

The similar robustness can also be found for the 2D EB non-Hermitian system. Details are shown in Supplementary Information S4. By considering that these flat distribution EB states have good robustness against spatial truncation and hopping disorder, we can construct ultrasensitive robust quantum sensors.

### Sensor model based on high-order EB states

**Fig. 3.**
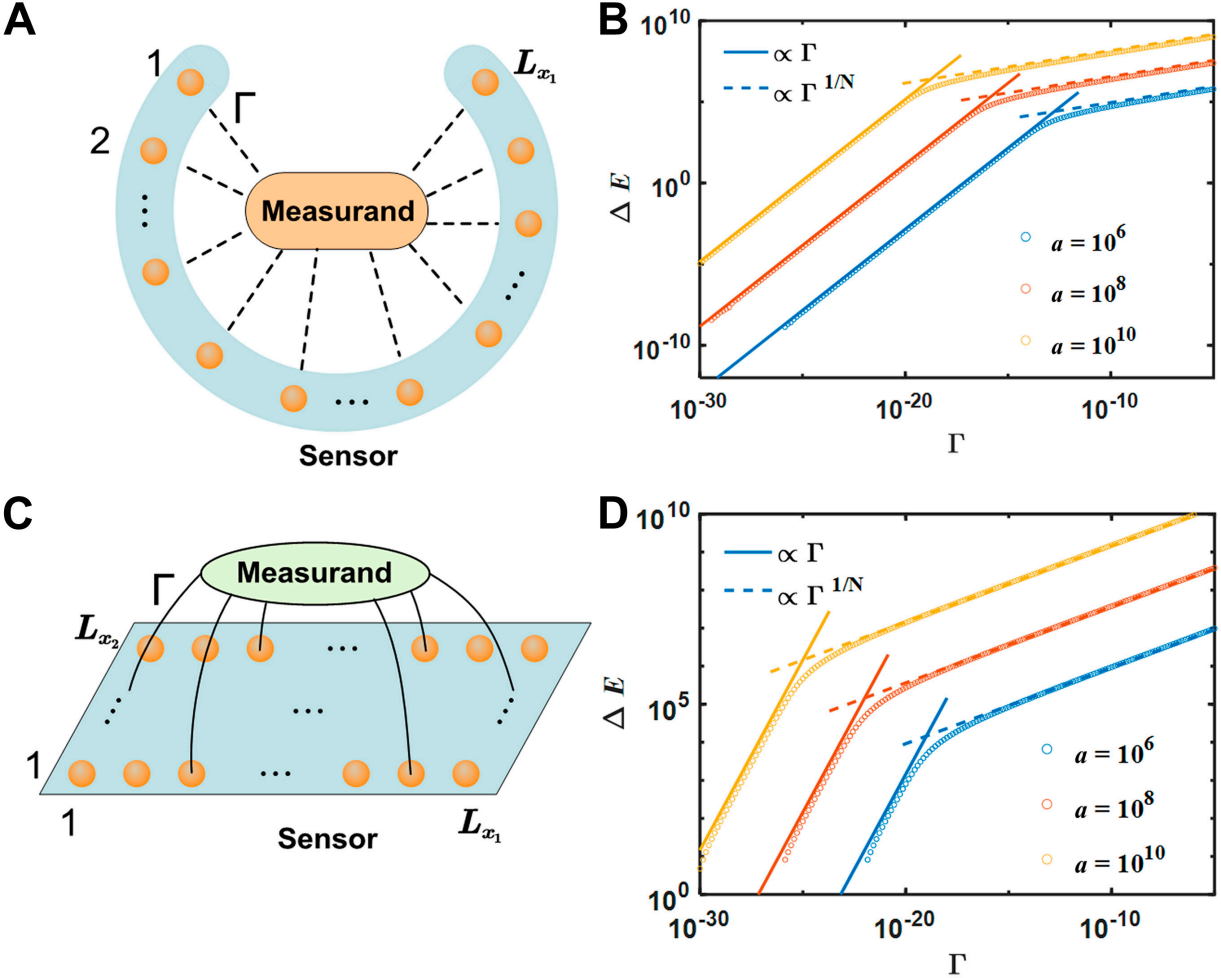
The sensor model based on the EB system. (A) Illustration of the sensor consisting of a 1D lattice with $L_{x_{1}}$ unit cells under open boundary conditions. The measurand connects between multiple sites with the coupling Γ. (B) For the 1D fifth-order system, the relationship between energy shift ∆E and the perturbation Γ with $L_{x_{1}}=100$ for several values of a. (C) Illustration of the sensor setup consisting of a 2D lattice with $L_{x_{1}}\times L_{x_{2}}$ unit cells under open boundary conditions. The measurand connects between multiple sites on the 2D lattice with the coupling Γ. (D) For the 2D fifth-order system, the relationship between energy shift ∆E and the perturbation Γ with $L_{x_{1}}=100,L_{x_{2}}=10$. In (B) and (D), blue, orange, and yellow circles represent the numerical results with $a=10^{6}$, $10^{8}$, and $10^{10}$, respectively.

In the following, we analyze the sensitivity of the constructed EB systems to external perturbations. For the 1D system, we consider the measurand Γ shown in Fig. 3A. When considering the connection between the first sublattice site of unit-cell $l_{1}$ (indexed by state $|l_{1},1\rangle$) and the last sublattice site of unit-cell $l_{2}$ (indexed by state $|l_{2},N\rangle$), the Hamiltonian of the external perturbations can be expressed as $∆P_{l_{1},l_{2}}=\Gamma \left(|l_{1},1\rangle \langle l_{2},N|+|l_{2},N\rangle \langle l_{1},1|\right..$ Thus, the measurand of connecting all the unit cells of the 1D chain with perturbation strength Γ in Fig. 3A is expressed as $∆P_{1D}=\sum_{l_{1}}\sum_{l_{2}\neq l_{1}}∆P_{l_{1},l_{2}}$. By performing numerical diagonalization, we obtain the eigenvalues $E\left(\Gamma \right)$ of the first EB states and the shift $∆E=E\left(\Gamma \right)-E_{0}$, where $E_{0}$ is the eigenvalues of the first EB states without perturbations.

In Fig. 3B, we plot numerical results of ∆E as a function of Γ with various a, which are represented by the circles. It is found that the curves of the relation between ∆E and Γ exhibit 2 distinct regimes. To better analyze the changing characteristics of ∆E, we perform systematic fitting for these numerical results. In fact, ∆E depends on several variables, including Γ, a, N, and L. Thus, through numerical calculations, we first take determined values for a and L, and discuss the functional relation of ∆E on Γ by numerical calculations. It is found that ∆E∝Γ at small ∆E regime and a power-law scaling $∆E\propto \Gamma^{1/N}$ at large ∆E regime. Then, with Γ and L fixed, we observe $∆E\propto a^{\left(N-1\right)/2}$ at small ∆E and $∆E\propto a^{\left(N-1\right)/N}$ at large ∆E. Finally, keeping Γ and a constant, we obtain the size scaling $∆E\propto L^{\theta_{1}\left(N-1\right)+1}$, where $\theta_{1}$ is determined by all of the nonreciprocal couplings and $\theta_{1}\approx 0.66$ provides excellent fitting results. These findings lead us to the comprehensive scaling relations:

$$∆E\propto \left\{\begin{array}{cc} \Gamma a^{\left(N-1\right)/2}{L_{x_{1}}}^{\theta_{1}\left(1-N\right)+1} & \text{small}\;∆E\\ \Gamma^{1/N}a^{\left(N-1\right)/N}{L_{x_{1}}}^{\theta_{1}\left(1-N\right)+1} & \text{large}\;∆E \end{array}\right..$$ (3)

The further detailed numerical fitting procedures and the first-order effect using perturbation theory are provided in Supplementary Information S5. The results in Eq. (3) also clearly demonstrates that the behavior of ∆E is governed by a and N. This can be attributed to 2 key factors: First, a can take significantly large values since $a=\frac{1}{2L_{x_{1}}}{∆}^{-4}$, where ∆ is a small offset. Second, the exponential dependence on N further amplifies the effect of a. For example, when taking $L_{x_{1}}=10$ and $\Gamma =10^{-15}$ with the same measurand via couplings of connecting all the unit cells, the shift of EB system is $∆E\approx 10^{-2}$, and the result of non-Hermitian Su–Schrieffer–Heeger (SSH) sensor [55] is $∆E\approx 10^{-14}$. Therefore, our sensing performance can display the improvement of 12 orders of magnitude when comparing with the non-Hermitian SSH sensor. When taking $L_{x_{1}}=20\;\text{and}\;\Gamma =10^{-15}$, our sensing performance can give the 10 orders of magnitude improvements.

The solid lines and dashed lines in Fig. 3B represent the fitted curves for 2 regimes, respectively. The agreements between numerical results and fitting curves are very well for a wide range of $\begin{array}{c} \Gamma . \end{array}$ Furthermore, the sensitivity $\begin{array}{c} S=\frac{\partial ∆E}{\partial \Gamma } \end{array}$ of the system can be calculated by the fitting expressions of ∆E. Compared with the recent proposed non-Hermitian SSH sensor [55], in which only edge states are used as the sensor unit, our proposed sensors can exhibit many advantages in sensing objects because all unit cells in the structure can be used as the sensor unit.

Similarly, we consider the measurand of connecting all the unit cells of the 2D EB system with perturbation strength Γ, which has been shown in Fig. 3C. Following the same fitting procedures, we can also establish the fitting relations that $∆E\propto \Gamma ,∆E\propto a^{\left(N-1\right)/2},$ and $\begin{array}{c} ∆E\propto L^{\theta_{2}\left(1-N\right)+1}= \end{array}$$\begin{array}{c} {\left(L_{x_{1}}\times L_{x_{2}}\right)}^{\theta_{2}\left(1-N\right)+1} \end{array}$ at small ∆E regime, while $∆E\propto \Gamma^{1/N},$ $∆E\propto a^{\left(N-1\right)/N},$ and $∆E\propto L^{\theta_{2}\left(1-N\right)+1}$ at large ∆E regime. The results also lead us to the comprehensive scaling relations:

$$\begin{array}{c} ∆E\propto \left\{\begin{array}{cc} \Gamma a^{\left(N-1\right)/2}L^{\theta_{2}\left(1-N\right)+1} & \text{small}\;∆E\\ \Gamma^{1/N}a^{\left(N-1\right)/N}L^{\theta_{2}\left(1-N\right)+1} & \text{large}\;∆E \end{array}\right., \end{array}$$ (4)

where $\theta_{2}$ represents the parameter for the 2D case. As revealed in Fig. 3D, good agreements between the fitting results and the numerical results are observed for the energy shift ∆E with perturbation Γ with N=5. Further details of the fitting process are described in Supplementary Information S5. The results are shown in Fig. 3D, where the circles denote the numerical results and lines denote the fitted results. Because all unit cells in the 2D structure can be used as the sensor unit, our system demonstrates superior performance ability. For example, when we take a perturbation $\Gamma =10^{-15}$, the corresponding $∆E\approx 10^{6}$ and $S\approx 10^{21}$ with $L_{x_{1}}=100$, $L_{x_{2}}=8,$ and $a=10^{6}$. This means that a very small perturbations can cause significant change of ∆E. That is to say, even minimal changes can be perceived by such a sensor.

Recent studies have shown that the signal-to-noise ratio (SNR) performance of EP-based sensors remains a controversial issue [61–65]. In these works, the SNR is defined as the standard deviation of the measured frequency shift, which is also employed in our study. The SNR at the EP is proportional to perturbations, which limits the detection accuracy enhancement of EP-based sensors. In contrast, our EB system exhibits stable SNR performance. This stability originates from the system’s operation at the EB state, where the energy is isolated and insensitive to common noise sources. The detailed analysis is provided in Supplementary Information S6, where we simulate the SNR under structural disorder and Gaussian noise, and show that the SNR can be stable even for strong nonreciprocal couplings, validating the robustness of the measurement.

Now, we explore how to experimentally construct the sensors mentioned above. Recent investigations have shown that classical electric circuits can be used to simulate various topological physics [49,52,53,56,59,66–72]. In the following, we explore the design of circuit networks to realize such sensors.

### Ultrasensitive circuit sensor based on high-order EB states

**Fig. 4.**
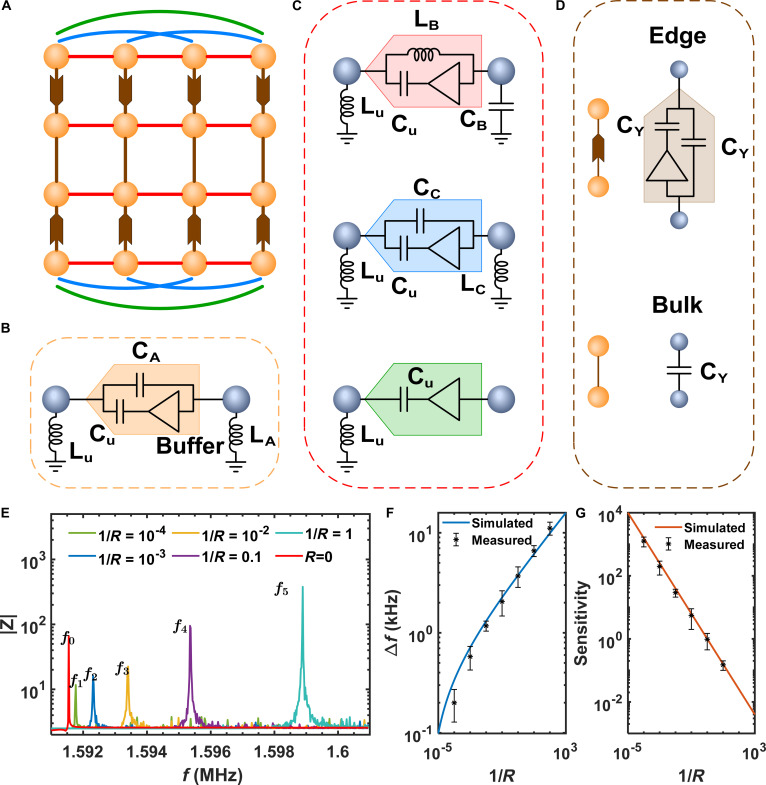
Realization of ultrasensitive sensor based on circuit networks. (A) Structural schematic diagram of the 2D EB circuit. The orange sphere represents the unit cell. The red, blue, and green solid lines represent the NN, second NN, and third NN nonreciprocal connections along the $x_{1}$ direction. The brown lines with triangles or rectangles represent couplings along the $x_{2}$ direction. (B) Detailed circuit structure of the unit cell, and the gray ball represents the circuit node. The orange arrow denotes the nonreciprocal connection consisting of capacitors ($C_{U}$ and $C_{A}$) and buffer. The grounding components $L_{U}$ and $L_{A}$ are used to eliminate the diagonal items. (C and D) Detailed circuit structure for the NN, second NN, and third NN connections along the $x_{1}$ direction, and the couplings along the $x_{2}$ direction. The circuit components are selected as $C_{U}=100\;\mathrm{uF},L_{u}=100\;\mathrm{pH},C_{A}=300\;\mathrm{pF},L_{A}=33\;\mathrm{uH},L_{B}=50\;\mathrm{uH},C_{B}=200\;\mathrm{pF},C_{C}=50\;\mathrm{pF},L_{C}=200\;\mathrm{uH},C_{Y}=10\;\mathrm{nF}$. (E) Circuit impedance spectrum with different values of R. Red, without perturbation and $f_{0}=1.5915\;\mathrm{MHz}$. The frequencies of peaks with perturbation are $\begin{array}{c} \;f_{1}=1.5917,\;f_{2}=1.5923,\;f_{3}=1.5934,\;f_{4}=1.5953,\;f_{5}=1.5989\;\mathrm{MHz} \end{array}$. Comparison of simulated and measured results for the frequency shift ∆f (F) and sensitivity (G) after adding the perturbation resistor.

Our designed circuit sensor for the 2D fifth-order Hamiltonian is schematically shown in Fig. 4A. The circuit in Fig. 4A includes orange spheres with $L_{x_{1}}\times L_{x_{2}}=4\times 4$, which correspond to the unit cells in Fig. 1A. Each orange sphere includes 5 nodes (gray spheres in Fig. 4B) corresponding to the sites within the unit cells. The connections between 5 nodes for the nonreciprocal couplings are achieved by capacitors ($C_{U}$ and $C_{A}$) and buffer, which has been shown in Fig. 4B. The purpose of the grounding inductances $L_{U}$ and $L_{A}$ is to eliminate the on-site energy offsets in the system. However, it should be noted that the offset at each lattice site is distinct, so these differing offsets cannot be fully eliminated merely by treating them as a translated spectrum.

For the above circuit of unit cell, it can be described by the Laplacian $J_{1}\left(\omega \right)$, where the elements are $J_{\left(l_{1},\;\alpha_{i}\right)\left(l_{1},\;\alpha_{i}+1\right)}=-\mathrm{i\omega}C_{U}$ and $J_{\left(l_{1},\;\alpha_{i}\right)\left(l_{1},\;\alpha_{i}-1\right)}=-\mathrm{i\omega}C_{A}$, and satisfy $C_{U}:C_{A}=a_{1}:b_{1}$. Correspondingly, appropriate values of the grounding components are also selected to make $C_{U}={\left(\omega_{0}^{2}L_{U}\right)}^{-1}$ and $C_{A}={\left(\omega_{0}^{2}L_{A}\right)}^{-1},$ with $\omega_{0}$ denoting the resonant frequency. Their specific nonreciprocal circuit connection diagrams for the NN, second NN, and third NN couplings are shown in Fig. 4C. The components are taken as $C_{B}:{\left(\omega^{2}L_{C}\right)}^{-1}:C_{D}=b_{2}:b_{3}:b_{4}$. As shown in Fig. 4D, the nonreciprocal coupling along the $x_{2}$ direction is realized by the combination of 2 capacitors $C_{Y}$ and one buffer, and the other reciprocal couplings is performed by the capacitor $C_{Y}$. The Laplacian $J\left(\omega \right)$ for the circuit network above corresponds to the Hamiltonian P. Detailed demonstration is given in Topolectric circuit design. The implementation for the circuit is provided in Supplementary Information S7.

Now, we explore the sensor effect of the circuit by tuning the resistor R as the perturbation, $\begin{array}{c} ∆J=R^{-1}\sum_{l_{1}}\sum_{l_{2}\neq l_{1}} \end{array}$$\begin{array}{c} \left(|l_{1},\;\alpha_{1}\rangle \langle l_{2},\;\alpha_{N}|+|l_{2},\;\alpha_{N}\rangle \langle l_{1},\;\alpha_{1}|\right) \end{array}$, and the impedance of the resistor 1/R corresponds to the perturbation strength Γ. We take the connection between the first node of the $l_{1}$ unit (indexed by the state $|l_{1},\;\alpha_{1}\rangle$) and the last node of the $l_{2}$ unit (indexed by the state $|l_{2},\;\alpha_{N}\rangle$). By connecting all the units with resistors, we observe the changes of the eigenvalues with the perturbation, which can be directly observed by measuring the impedance response [66]. The red line in Fig. 4E shows the frequency of the EB flat state without the perturbation. The other colored lines represent the experimental measured spectra of the impedance response with various perturbations. It is observed clearly that the shift of resonant frequency corresponds to the peak impedance shifts. Details are given in The impedance measurement on the circuit.

The black dots in Fig. 4F show the measured results for the frequency shifts of impedance peaks ∆f as a function of the perturbation (1/R), while the blue line is the fitted result. The agreements between the experimental and fitted results are very well. With the choice of electric components, the frequency shift of impedance satisfies with the perturbation as $∆f\propto {\left(1/R\right)}^{1/5}$, which is the same to the results in the lattice model in the large eigenvalue-shift case. In Fig. 4G, we provide the sensitivity results based on the resonance frequency. The black dots represent the experimental results, while the orange line is the fitted sensitivity that is described as $S_{\mathrm{cir}}\propto {\left(1/R\right)}^{4/5}$. It is also the same to the results in the lattice model. Furthermore, the intrinsic robustness of EB states consequently ensures the stable sensing performance, with detailed analysis provided in Supplementary Information S8. In addition, due to the intrinsic robustness of the long-range coupling design and effective suppression of circuit noise through precision components and active compensation, the SNR of the designed circuit remains very stable. Detailed noise analysis is provided in Supplementary Information S9.

**Fig. 5.**
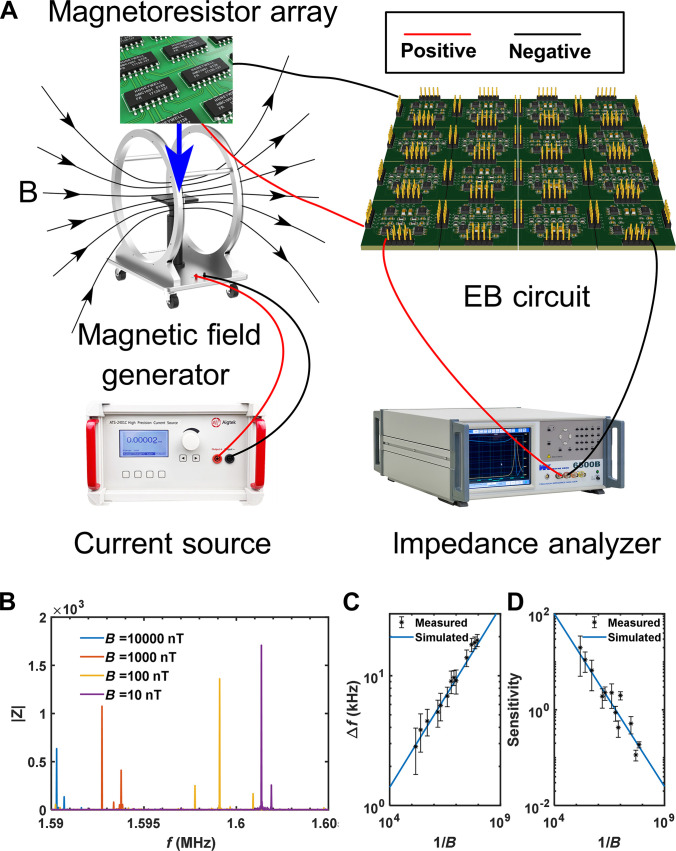
The ultrasensitive detection of the weak magnetic field. (A) Schematic diagram of the experimental device, including a magnetic field generator which generates the magnetic field as ‘B’, magnetoresistor, EB sensor circuit, and impedance analyzer for the measurement. (B) Relationship between the circuit impedance and frequency under different external magnetic fields. Comparison of simulated and measured results for the frequency shift ∆f (C) and sensitivity (D) after adding different external magnetic fields.

As a practical application example, now we explore how to use our designed circuit sensor to measure magnetic fields. In general, the magnetic field can have an impact on a region, and therefore, the signals on the region can be accumulated to be detected. Not like the non-Hermitian SSH chain sensor [55] whose working areas are just 2 ends of chain, our proposed EB circuit sensor can capture the signal on the whole surface of circuit network. Figure 5A shows the schematic diagram of the experimental device, including the magnetic field generation, the magnetoresistance chip, the designed EB circuit, and the impedance measurement part.

The weak magnetic field to be measured is generated by the Helmholtz coil, and the required strength of the weak magnetic field can be accurately controlled by adjusting the high-precision current source connected to the coil. The magnetoresistance chip is made of a thin layer of nickel–iron film and arranged as a resistor. In our experiment, many magnetoresistance chips serve as the perturbation resistors. After applying different external magnetic fields B,the resistanceR also changes in a proportional relationship due to the magnetoresistance effect, realizing the transformation from the measured physical quantity (magnetic field strength) to the circuit quantity. In addition, in order to eliminate the influence of the geomagnetic field, we compensate by biasing the current to generate a reverse magnetic field. Then, due to the high sensitivity and strong amplification effect of our sensor circuit, the weak change of the resistance causes a significant shift of the impedance peak in the circuit, which corresponds to the significant shift of the eigenvalue of the EB state under the perturbation. Finally, the shift of the impedance peak can be measured by connecting the EB circuit and impedance analyzer through positive and negative electrodes. Details are provided in Supplementary Information S10.

Figure 5B displays the impedance spectra at the first node of the first unit cell under magnetic fields of the magnetic field $B=10,10^{2},10^{3},$and $10^{4}\;\mathrm{nT}$ (purple, yellow, orange, and blue lines, respectively). The rationale for selecting this node is that the distribution of the EB states at this location is flat, which accurately reflects the flat characteristics of the EB states. It can be clearly seen that the impedance spectrum measured under different external magnetic fields varies with the frequency, and the specific relationship between ∆f and B is shown in Fig. 5C. Figure 5D also shows the results of relationship between sensitivity and B. It is found that the experimental results based on the magnetic field are in good agreement with the theoretical results. Therefore, the EB sensor circuit we designed has been successfully applied to the ultrasensitive detection of weak magnetic fields. Little difference between points and lines comes from the external interference magnetic fields and loss of electric components. Moreover, the noise of resistor is also considered. In Supplementary Information S9, we have studied the effect of different levels of noise, the robustness of the sensing mechanism ensures performance stability under different fluctuations.

At end, we would like to emphasize that the designed circuit sensors are based on printed circuit board (PCB) platforms. In fact, the corresponding on-chip ultrasensitive sensing systems can also be realized by using a complementary metal-oxide semiconductor (CMOS) process technology [53,73]. The chips using CMOS technology can show the advantages as it is easily configurable, the parasitic effect is reduced enormously, and the operating frequency of the mode can also be increased. Thus, the sensitivity of circuit sensors based on CMOS technology can be improved by orders of magnitude compared to those based on PCB platform. That is, the detection limit of the magnetic field in Fig. 5 is *nT*. As a comparison, when the CMOS technology is used, weak magnetic signals as small as *fT* are expected to be detected.

Here, we have demonstrated magnetic field sensing using the magnetoresistor. Indeed, the core sensing mechanism is not limited to magnetic fields, and the same platform can be directly adapted to sense any physical quantity convertible into a change in circuit, such as displacement, angle, or liquid level, by connecting corresponding capacitive transducers. In addition, our results reveal that the circuit serves as a powerful and flexible platform for realizing the targeted quantum Hamiltonian. This enables precise access to the relevant eigenstates of the system, thereby facilitating experimental demonstration of the sensing enhancement predicted by quantum theory.

## Discussion

The sensing mechanism proposed in this work can be further analyzed by comparing it with other advanced sensing schemes, particularly EP-based sensors, topological quantum sensors, and conventional quantum sensors. EP-based sensors exploit the spectral singularity at non-Hermitian degeneracies, and the frequency splitting for an *n*th−order EP is generically proportional to the *n*th root of perturbation strength $\begin{array}{c} \Gamma \end{array}$. However, their practical utility is hindered by the amplification of noise due to extreme non-orthogonality of modes (divergent Petermann factor) [9,10]. For the non-Hermitian topological chain sensors, the energy shift of a boundary mode scales exponentially with system size. However, its exponential sensing performance often requires the measurand connecting between the 2 ends of chain [55]. For the conventional quantum sensor [74], although it shows unprecedented sensitivity and spatial resolution, the decoherence time is very short, which leads to increased control difficulty. As a comparison, our proposed sensor shows the stable SNR, and the detected areas of sensor are not limited to the ends of system. In addition, our sensor does not suffer from the decoherence and is able to continue working for long times.

In this work, we have theoretically disclosed a new class of robust quantum states and higher-order EB states in exceptional systems. These quantum states can be engineered by tuning the nonreciprocal couplings of the system. Especially, we can design quantum states with flat spatial distributions for both 1D and 2D systems. Compared to the second-order EB states, our designed high-order EB states exhibit more robust against disorders. Based on such states, we have constructed a novel class of quantum sensors. We have also demonstrated that such sensors not only have unprecedented sensitivity but also are robust against disorders. They have been proven to have significant advantages compared to existing quantum sensors. Furthermore, we have found that higher-order EB states can also exist in classical systems. We have designed and fabricated corresponding circuit network and integrated circuit sensors to experimentally observe the phenomena. Taking magnetic field detection as an example, we have demonstrated the effectiveness of our designed circuit sensor. Therefore, our proposed new quantum sensing scheme is expected to have widely potential applications in various fields.

## Methods

### Topolectric circuit design

Topological circuits are an emerging synthetic topological material platform that allows any tight-binding model of any coupling range or dimension to be realized. In such circuits, the behavior of the system is governed by fundamental electrical laws, among which Kirchhoff's law plays a central role. Specifically, based on Kirchhoff’s law, the voltage and current at the node have the relation:

$$I_{m}=\sum_{n\neq m}Y_{\mathrm{mn}}\left(V_{m}-V_{n}\right)+w_{m}V_{m},$$ (5)

where $I_{m}$ and $V_{m}$ are the input current and electrical voltage at node m. According to the conservation of current, $I_{x}$ is equal to the total current flowing out of node m toward all other nodes n linked by the admittance $Y_{\mathrm{mn}}$, plus the current flowing from node m to the ground through the path with the admittance $w_{m}$. For a single capacitive, resistive, and inductive component, the admittance are taken as iωC,1/R,and1/iωL, respectively.

In the circuit, the critical design consideration involves the realization of nonreciprocal coupling mechanisms. Here, the fundamental nonreciprocal connection is shown in Fig. 4B. The black triangle represents the buffer, which play the role of voltage follower. By connecting capacitor *C_U_* in series with the buffer, the other capacitance *C_A_* in parallel is added. Based on the characteristic that buffer can block the input current while keeping the output voltage stable, we get the following equation by carrying out Kirchhoff’s law on the circuit node 1 (left) and node 2 (right):

$$\begin{array}{c} I_{1}=\mathrm{i\omega}C_{A}\left(V_{1}-V_{2}\right)\\ I_{2}=\mathrm{i\omega}C_{A}\left(V_{2}-V_{1}\right)+\mathrm{i\omega}C_{U}\left(V_{2}-V_{1}\right) \end{array}.$$ (6)

Equation (6) can be reexpressed in a matrix form as:

$$\left(\begin{array}{c} I_{1}\\ I_{2} \end{array}\right)=\left(\begin{array}{cc} \mathrm{i\omega}C_{A} & -\mathrm{i\omega}C_{A}\\ \mathrm{i\omega}\left(C_{U}+C_{A}\right) & \mathrm{i\omega}\left(C_{U}+C_{A}\right) \end{array}\right)\left(\begin{array}{c} V_{1}\\ V_{2} \end{array}\right).$$ (7)

It can be seen that the conductance matrix is a non-Hermitian matrix, where 2 off-diagonal elements of $C_{A}$ and $C_{U}+C_{A}$ represent effective values of the connecting capacitor between 2 nodes, and the nonreciprocal capacitance with the value being $C_{A}$ and $C_{U}+C_{A}$ can be achieved.

Similarly, other nonreciprocals can be realized in circuit and schematically depicted in Fig. 4C and D. They are all realized by buffers, capacitors, and inductors, and the corresponding grounding components are also different. In the $x_{2}$ direction, the circuits for realizing 2 kinds of couplings are shown in Fig. 4D. The nonreciprocal coupling is realized by the combination of 2 capacitors $C_{Y}$ and one buffer, and the other reciprocal couplings is performed by the capacitor $C_{Y}$. For such a circuit network above, we can theoretically derive its circuit Laplacian $J\left(\omega \right)$. In our study, the currents flowing into each node of circuit can be written as:

I1=∑j=03iωCUV1−V2+5j+4iωCYV21I2=∑j=03iωCUV2−V3+5j+iωCAV2−V1+1iωLBV2−V6+iωCCV2−V11+4iωLU+1iωLA+iωCB+1iωLCV2+iωCYV22I3=∑j=03iωCUV3−V4+5j+iωCAV3−V2+1iωLBV3−V7+iωCCV3−V12+4iωLU+1iωLA+iωCB+1iωLCV3+iωCYV23…I80=iωCCV80−V69+1iωLBV80−V74+iωCAV80−V79+1iωLA+iωCB+1iωLCV80+2iωCYV60 (8)

Equation (8) can be recast into a matrix form:

I1I2I3⋮I80=iωY1−CU0⋯0−CAY2−CU⋯00−CAY3⋯0⋮⋮⋮⋱⋮000⋯Y80V1V2V3⋮V80, (9)

where $\begin{array}{c} \begin{array}{c} Y_{1}=4C_{U}-\frac{4}{\omega^{2}L_{U}},Y_{2}=4C_{U}+C_{A}-\frac{1}{\omega^{2}L_{B}}+C_{C}-\frac{4}{\omega^{2}L_{U}}-\frac{1}{\omega^{2}L_{A}}+ \end{array} \end{array}$$\begin{array}{c} C_{B}-\frac{1}{\omega^{2}L_{C}},\ldots ,Y_{80}=C_{A}-\frac{1}{\omega^{2}L_{B}}+C_{C}+\frac{1}{\mathrm{i\omega}L_{A}}+\mathrm{i\omega}C_{B}+\frac{1}{\mathrm{i\omega}L_{C}} \end{array}$ are the diagonal terms that are determined by all the components connected to the corresponding node and the grounding components. The circuit Laplacian can be obtained as:

$$J\left(\omega \right)=\mathrm{i\omega}\left(\begin{array}{ccccc} Y_{1} & -C_{U} & 0 & \cdots & 0\\ -C_{A} & Y_{2} & -C_{U} & \cdots & 0\\ 0 & -C_{A} & Y_{3} & \cdots & 0\\ \vdots & \vdots & \vdots & \ddots & \vdots \\ 0 & 0 & 0 & \cdots & Y_{80} \end{array}\right).$$ (10)

If the parameters are chosen as $\begin{array}{c} C_{U}:C_{A}:-{\left(\omega_{0}^{2}L_{B}\right)}^{-1}:C_{C}: \end{array}$$\begin{array}{c} C_{Y}=a:b_{1}:b_{2}:b_{3}:y_{0},\omega =\omega_{0} \end{array}$ and $\begin{array}{c} C_{U}=\frac{1}{\omega_{0}^{2}L_{U}},C_{A}=\frac{1}{\omega_{0}^{2}L_{A}},C_{B}=\frac{1}{\omega_{0}^{2}L_{B}}, \end{array}$$\begin{array}{c} C_{C}=\frac{1}{\omega_{0}^{2}L_{C}} \end{array}$, i.e. $Y_{1}=Y_{2}=\ldots =Y_{80}=0$, the circuit Laplacian $J\left(\omega \right)$ has a good correspondence to the lattice model Hamiltonian at the resonance frequency $\omega_{0}$ that $J\left(\omega_{0}\right)\propto P$.

### The impedance measurement on the circuit

In the circuit, we usually observe the characteristics of the system through the impedance response $Z_{\mathrm{ab}}\left(\omega \right)=\frac{V_{a}-V_{b}}{I}$, which is the ratio of voltage between nodes a and b due to the magnitude of the current that enters at a and leaves at b. Here, the 2-point impedance of node a and b reads:

$$Z_{\mathrm{ab}}=\sum_{n}\frac{{\left|\varphi_{n,a}-\varphi_{n,b}\right|}^{2}}{E_{\mathrm{cir},n}},$$ (11)

where $E_{\mathrm{cir},n}$ and $\varphi_{n,a}-\varphi_{n,b}$ denote the eigenvalue and the difference between the amplitudes of the nth admittance eigenmode of circuit Laplacian J. Based on the above expression of the impedance, it can be observed that $Z_{\mathrm{ab}}$ becomes significantly large when there exists a finite density of nontrivial eigenmodes with small eigenvalues $E_{\mathrm{cir},n}$. For each eigenvalue $E_{\mathrm{cir},n}\left(\omega \right)$, there corresponds a resonant frequency $\tilde{\omega_{n}}$ at which $E_{\mathrm{cir},n}\left(\tilde{\omega_{n}}\right)$ attains a very small value ε. Consequently, this gives rise to a dominant term in the impedance, leading to the approximate relation $Z\left(\tilde{\omega_{n}}\right)\approx \frac{1}{\varepsilon }{\left|\varphi_{n,1}\right|}^{2}$. Given the existence of multiple eigenvalues $E_{\mathrm{cir},n}$, a series of distinct resonant frequencies $\tilde{\omega_{n}}$ can be identified. Furthermore, any shift in the eigenvalues directly corresponds to a shift in these resonant frequencies. Therefore, for the resonance frequency $\omega_{0}$ of the circuit Laplacian J without perturbation, there is a peak in impedance spectrum $Z\left(\omega \right)$, and the frequency shifts of this impedance peak represent the energy shift of the first EB mode.

## Data Availability

The data that support the plots within this paper are available from the corresponding authors upon reasonable request.
