# Supplementary material for: Ultrasensitive Quantum Sensors Based on High-Order Exceptional Bound States
Source: Research (Wash D C). 2026 Feb 5;9:1091. doi: 10.34133/research.1091 (PMC12873468; doi:10.34133/research.1091)
Supplement: Supplementary 1 — Supplementary Information S1 to S10 Figs. S1 to S13 Tables S1 and S2 [file research.1091.f1.docx]

**Supplementary Information for**

**Ultra-sensitive quantum sensors based on high-order exceptional bound states**

Shaohui Liu^*^, Tian Chen^*^, Deyuan Zou and Xiangdong Zhang^+^

*Key Laboratory of advanced optoelectronic quantum architecture and measurements of Ministry of Education, School of Physics, Beijing Institute of Technology, 100081, Beijing, China*

**These authors contributed equally to this work. ^+$^Author to whom any correspondence should be addressed. E-mail:* [*zhangxd@bit.edu.cn*](mailto:zhangxd@bit.edu.cn)*,* [*chentian@bit.edu.cn*](mailto:chentian@bit.edu.cn)

Supplementary Information S1. The details for selecting the functional form of $f\left( k_{x_{1}} \right)$ and the derivation process of coefficients for couplings.

Supplementary Information S2. Detailed calculation process of eigenvalues and eigenstates.

2.1 One-dimensional case

2.2 Two-dimensional case

Supplementary Information S3. The construction of the high dimensional EB systems.

Supplementary Information S4. Robustness of EB states for 1D and 2D system.

Supplementary Information S5. Details of the fitting process under the perturbation.

5.1 One-dimensional case

5.2 Two-dimensional case

Supplementary Information S6. Calculation process of signal-to-noise ratio.

Supplementary Information S7. Printed Circuit Board (PCB) fabrications and measured results.

Supplementary Information S8. Robustness of EB circuit.

Supplementary Information S9. Noise analysis of impedance spectra of designed circuits.

Supplementary Information S10. Detailed design methods and properties of the magnetic field measurement experiment.

**Supplementary Information S1. The details for selecting the functional form of** $\boldsymbol{f}\left( \boldsymbol{k}_{\boldsymbol{x}_{\boldsymbol{1}}} \right)$ **and the derivation process of coefficients for couplings.**

This section presents the reason for selecting the functional form of $f\left( k_{x_{1}} \right)$. As the functional form of $f\left( k_{x_{1}} \right)$ critically affects the system by determining the coupling strength distribution in the two-dimensional (2D) exceptional bound (EB) system Hamiltonian $P_{2D}$, we must analyze it in the specific expression of $P_{2D}$. This requires first establishing how the $P_{2D}$ emerges from the given 2D EP system $H_{0}$, which takes the form:

$\begin{aligned} H_{0}=H_{1}\left( k_{x_{1}} \right)-2y_{0}\cos k_{x_{2}}\mathbb{I}_{N\times N} \#\left( S1 \right) \end{aligned}$

where $H_{1}\left( k_{x_{1}} \right)$ is the 1D system containing the $N$th-order exceptional point (EP), and all these 1D lattices are connected between the nearest neighboring 1D EP lattices along the $x_{2}$-direction. For simplicity, we consider the most elementary form of EP system $H_{1}\left( k_{x_{1}} \right)$, which expressed as:

$$\begin{aligned} H_{1}\left( k_{x_{1}} \right)=\left( \begin{matrix} 0 & 1 & & & 0 \\ f\left( k_{x_{1}} \right) & \ddots& \ddots& & \\ & \ddots& \ddots& \ddots& \\ & & \ddots& \ddots& 1 \\ 0 & & & f\left( k_{x_{1}} \right) & 0 \end{matrix} \right)_{N\times N} .\#\left( S2 \right) \end{aligned}$$

In our discussion, the form of $f\left( k_{x_{1}} \right)$​ is constrained that $f\left( k_{x_{1}} \right)\to k_{x_{1}}^{B}$ where $B>0$. In this way, the EP emerges at the value $k_{x_{1}}=0$. Therefore, $H_{1}\left( 0 \right)$ is defective, possessing only one right eigenvector $\left( 1,0,\ldots,0 \right)^{T}$, and holds the $N$th-order EP. The above expression clearly demonstrates that the specific form of $f\left( k_{x_{1}} \right)$​ entirely governs the properties of $H_{0}$​, thereby influencing the couplings in $P_{2D}$.

Based on the above formulation of $H_{0}$, we can construct the EB system Hamiltonian $P_{k}=\frac{1}{2}\left( \mathbb{I+}\frac{H_{0}}{E_{0}} \right)$, where $E_{0}$ is the ground state of $H_{0}$. And the form of $P_{k}$ in real space after inverse Fourier transform can be expressed as follows:

$$\begin{aligned} P=\sum_{\boldsymbol{k}} \left| \boldsymbol{k} \right\rangle\left\langle\boldsymbol{k} \right|\otimes P_{k} \#\left( S3 \right) \end{aligned}$$

where $\left| \boldsymbol{k} \right\rangle=\frac{1}{\sqrt{L}}\sum_{\boldsymbol{x}} e^{i\boldsymbol{x}\cdot\boldsymbol{k}}\left| \boldsymbol{x} \right\rangle$ is the basis vector in momentum-space, $\left| \boldsymbol{x} \right\rangle$ is basis vector for unit cell of real space and $L=L_{x_{1}}\times L_{x_{2}}$ is the scales of the system. Here, $\boldsymbol{k}$ takes discrete values within the two-dimensional Brillouin zone, and we need to consider $\boldsymbol{k}^{\boldsymbol{'}}\boldsymbol{=k+}\boldsymbol{\Delta}$ to avoid the singularity caused by the degeneracy at the EP.

Then, under the open boundary condition (OBC) with boundary correction, the Hamiltonian in real space $P_{2D}$ of Eq. (S3) can be expressed as:

$$\begin{aligned} P_{2D}=\left( \begin{matrix} P_{1D} & 2y_{0}\mathbb{I} & 0 & 0 & \cdots& 0 \\ y_{0}\mathbb{I} & P_{1D} & y_{0}\mathbb{I} & 0 & \cdots& 0 \\ 0 & y_{0}\mathbb{I} & P_{1D} & y_{0}\mathbb{I} & \cdots& \vdots\\ 0 & 0 & y_{0}\mathbb{I} & P_{1D} & \ddots& 0 \\ \vdots& \vdots& \vdots& \ddots& \ddots& y_{0}\mathbb{I} \\ 0 & 0 & \cdots& 0 & 2y_{0}\mathbb{I} & P_{1D} \end{matrix} \right) \#\left( S4 \right) \end{aligned}$$

with

$$\begin{aligned} P_{1D}=\left( \begin{matrix} \boldsymbol{A}_{1} & \boldsymbol{A}_{2} & \boldsymbol{A}_{3} & \cdots& \boldsymbol{A}_{L_{x_{1}}} \\ \boldsymbol{A}_{2} & \boldsymbol{A}_{1} & \boldsymbol{A}_{2} & \cdots& \boldsymbol{A}_{L_{x_{1}}-1} \\ \boldsymbol{A}_{3} & \boldsymbol{A}_{2} & \boldsymbol{A}_{1} & \cdots& \boldsymbol{A}_{L_{x_{1}}-2} \\ \vdots& \vdots& \ddots& \ddots& \vdots\\ \boldsymbol{A}_{L_{x_{1}}} & \boldsymbol{A}_{L_{x_{1}}-1} & \boldsymbol{A}_{L_{x_{1}}-2} & \cdots& \boldsymbol{A}_{1} \end{matrix} \right), \#\left( S5 \right) \end{aligned}$$

where the lattice structure described by $P_{2D}$ is identical to the structure of Fig.1**a** in the main text. $P_{1D}$ represents couplings among the lattice along the $x_{1}$-direction of $P_{2D}$. Here, all of the block matrix in$\mathrm{Eq}$. (S5) from $\boldsymbol{A}_{1}$ to $\boldsymbol{A}_{L_{x_{1}}}$ are $N\times N$ tridiagonal Toeplitz matrix. These block matrices are all in the form of tridiagonal matrices:

$$\begin{aligned} \boldsymbol{A}_{l}=-\left( \begin{matrix} \delta_{l,1} & a_{l} & & & 0 \\ b_{l} & \ddots& \ddots& & \\ & \ddots& \ddots& \ddots& \\ & & \ddots& \ddots& a_{l} \\ 0 & & & b_{l} & \delta_{l,1} \end{matrix} \right)_{N\times N} ,\#\left( S6 \right) \end{aligned}$$

where $l=1,\ldots,L_{x_{1}}$ is the index of unit-cells, $a_{l}=\frac{1}{2L_{x_{1}}}\sum_{k_{x_{1}}^{'}} \left[ f\left( k_{x_{1}} \right) \right]^{-1/2}e^{ik_{x_{1}}^{'}\left( l-1 \right)}, b_{l}=\frac{1}{2L_{x_{1}}}\sum_{k_{x_{1}}^{'}} \left[ f\left( k_{x_{1}} \right) \right]^{1/2}e^{ik_{x_{1}}^{'}\left( l-1 \right)}$ are the strength of the non-reciprocal couplings and $\delta$ is Dirac function.

As discussed previously, $f\left( k_{x_{1}} \right)\to k_{x_{1}}^{B}$ as $k_{x_{1}}\to0$. Consequently, when $\boldsymbol{\Delta}\to0$, $a_{l}$​ exhibits a divergent behavior, whereas $b_{l}$ rapidly converges. To ensure numerical stability for small $\boldsymbol{\Delta}$, it is essential that $a_{l}$ remains approximately stable near a well-defined limiting value, and $b_{l}$ maintains sufficiently fast convergence. Based on these limitations, we systematically evaluate and select among various valid ansatze that are available for $f\left( k_{x_{1}} \right)$, and finally we adopt $f\left( k_{x_{1}} \right)=[2\left( 1-\cos k_{x_{1}} \right)]^{4}$, which yields the most local Fourier coefficients. Although alternative ansatze are possible, such as $f\left( k_{x_{1}} \right)=\left( 1-e^{ik_{x_{1}}} \right)^{B}$ or $\sin^{B} k$, the ansatz $\left( 1-e^{ik_{x_{1}}} \right)^{B}$ yields non-local coefficients $b_{l}$, while ansatz $\sin^{B} k$ produces the staggered $a_{l}$. In contrast, only the ansatz $[2\left( 1-\cos k_{x_{1}} \right)]^{B}$ possesses the most local Fourier coefficients $a_{l}$ and $b_{l}$, as shown in Fig. S1. The specific choice of $[2\left( 1-\cos k_{x_{1}} \right)]^{4}$ is justified by the fact that that $B=4$ represents the minimal exponent ensuring maximally identical coefficients $a_{l}$.


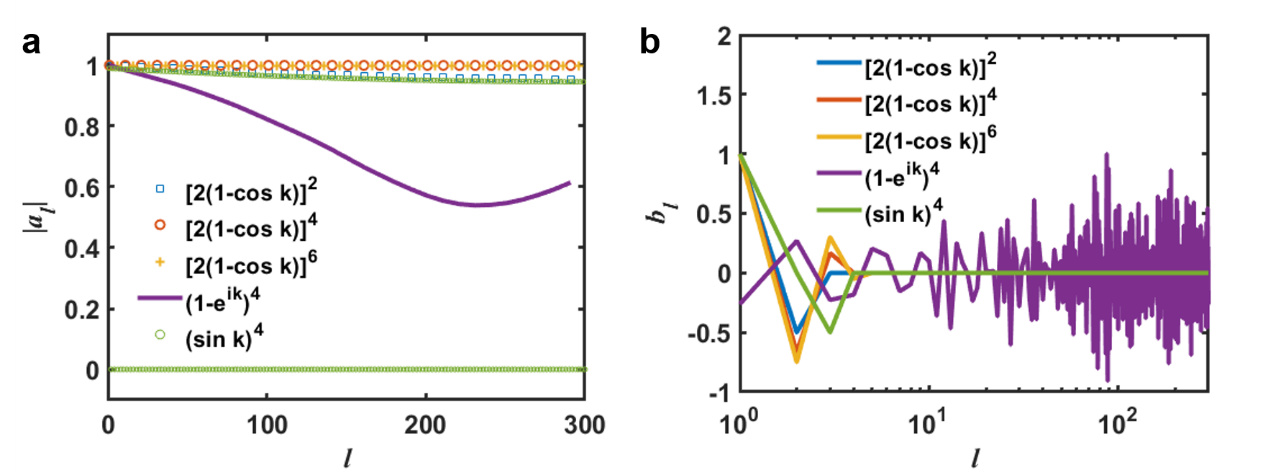


**Figure S1.** Comparisons between $a_{l}$ and $b_{l}$ of various ansatz. (a) and (b) respectively depict $a_{l}$ and $b_{l}$ (both normalized to have a maximum value of 1) for $f\left( k_{x_{1}} \right)$ defined by $f\left( k_{x_{1}} \right)=\left( 1-e^{ik} \right)^{4}$ or $\sin^{4} k$, and $f\left( k_{x_{1}} \right)=[2\left( 1-\cos k \right)]^{B}$ with $B=2,4,6$.

Then, we present a detailed theoretical derivation of $a_{l}$ and $b_{l}$ for the selected ansatz $[2\left( 1-\cos k_{x_{1}} \right)]^{4}$ by performing the Fourier transform under the assumption that $\Delta$ is a small parameter. From the above results, we obtain that $a_{l}=\frac{1}{2L_{x_{1}}}\sum_{k_{x_{1}}^{'}} \frac{e^{ik_{x_{1}}^{'}\left( l-1 \right)}}{\left( 1-\cos k_{x_{1}}^{'} \right)^{2}}=\frac{1}{2L_{x_{1}}}\sum_{k_{x_{1}}^{'}} \frac{e^{ik_{x_{1}}^{'}\left( l-1 \right)}}{16\sin^{4} k_{x_{1}}^{'}/2}$. A dominant contribution arises from the $\sin k^{'}/2=0$ terms in the summation. Subsequent series expansion yields the relation:

$$\begin{aligned} a_{l}\approx\frac{1}{2L_{x_{1}}}\Delta^{-4}+i\frac{\left( l-1 \right)}{2L_{x_{1}}}\Delta^{-3}+\frac{1-3\left( l-1 \right)^{2}}{12L_{X}}\Delta^{-2}, \#\left( S7 \right) \end{aligned}$$

which is mainly decided by $\Delta^{-4}$ when $l$ is relatively small. When taking $\Delta={10}^{-2}$, $a_{1}=\cdots=a_{L_{x_{1}}}=a\approx{10}^{6}$.

Besides, when considering $b_{l}$, we can further apply the identity $\sin^{4} x=\frac{3}{8}-\frac{1}{2}\cos2x+\frac{1}{8}\cos4x$ to calculate $b_{l}$ as follows:

$$b_{l}=\frac{8}{L_{x_{1}}}\sum_{l=1}^{L_{x_{1}}} \sin^{4} \left( \frac{k_{x_{1}}^{'}}{2} \right)e^{ik_{x_{1}}^{'}\left( l-1 \right)}$$

$$\begin{aligned} =\frac{8}{L_{x_{1}}}\left[ \frac{3}{8}S_{0}-\frac{1}{2}S_{1}+\frac{1}{8}S_{2} \right],\#\left( S8 \right) \end{aligned}$$

where $S_{0}=\sum_{l=1}^{L_{x_{1}}} e^{ik_{x_{1}}^{'}\left( l-1 \right)}=\delta_{l,1}L_{x_{1}}$, $S_{1}=\sum_{l=1}^{L_{x_{1}}} \cos k_{x_{1}}^{'}e^{ik_{x_{1}}^{'}\left( l-1 \right)}=\delta_{l,2}{L_{x_{1}}}/2$ and $S_{2}=\sum_{l=1}^{L_{x_{1}}} \cos2k_{x_{1}}^{'}e^{ik_{x_{1}}^{'}\left( l-1 \right)}=\delta_{l,3}{L_{x_{1}}}/2$. Then, we can obtain that $b_{1}=3, b_{2}=-2,b_{3}=0.5,b_{4}\approx\ldots\approx b_{L_{x_{1}}}\approx0$.

For the one-dimensional (1D) case, the Hamiltonian follows a form analogous to the above discussion, as given in Eq. (S5), which corresponds to the lattice along the $x_{1}$-direction of $P_{2D}$. The specific coupling terms are identical to those described in Eqs. (S7) and (S8).

**Supplementary Information S2. Detailed calculation process of eigenvalues and eigenstates.**

**2.1 One-dimensional case**

In this section, we derive the analytical expressions of the eigenvalues and eigenstates of the 1D EB system $P_{1D}$ by solving the eigen-equation $P_{1D}\left| \psi\right\rangle=E\left| \psi\right\rangle$, and define $\psi_{i,j}$ as the element of the $j$th sublattice of the $i$th unit cell of $\left| \psi\right\rangle$. Each term of the eigenequation is expanded as following. When considering the first sublattice sites of all the unit-cells, we have

$$\begin{aligned} \left\{ \begin{aligned} a\sum_{i=1}^{L_{x_{1}}} \psi_{i,2}=E\psi_{1,1} \\ a\sum_{i=1}^{L_{x_{1}}} \psi_{i,2}=E\psi_{2,1} \\ \vdots\\ a\sum_{i=1}^{L_{x_{1}}} \psi_{i,2}=E\psi_{L_{x_{1}}-1,1} \\ a\sum_{i=1}^{L_{x_{1}}} \psi_{i,2}=E\psi_{L_{x_{1}},1} \end{aligned} \right.,\#\left( S9 \right) \end{aligned}$$

and we can obtain that

$$\begin{aligned} \psi_{1,1}=\psi_{2,1}=\ldots=\psi_{L_{x_{1}}-1,1}=\psi_{L_{x_{1}},1}=\alpha. \#\left( S10 \right) \end{aligned}$$

Similarly, when considering the second sublattice sites of all the unit-cells, we have

$$\begin{aligned} \left\{ \begin{aligned} a\sum_{i=1}^{L_{x_{1}}} \psi_{i,3}+\left( b_{1}\alpha+b_{2}\alpha+b_{3}\alpha\right)=E\psi_{1,2} \\ a\sum_{i=1}^{L_{x_{1}}} \psi_{i,3}+\left( b_{2}\alpha+b_{1}\alpha+b_{2}\alpha+b_{3}\alpha\right)=E\psi_{2,2} \\ a\sum_{i=1}^{L_{x_{1}}} \psi_{i,3}+\left( b_{3}\alpha+b_{2}\alpha+b_{1}\alpha+b_{2}\alpha+b_{3}\alpha\right)=E\psi_{3,2} \\ \vdots\\ a\sum_{i=1}^{L_{x_{1}}} \psi_{i,3}+\left( b_{3}\alpha+b_{2}\alpha+b_{1}\alpha+b_{2}\alpha+b_{3}\alpha\right)=E\psi_{L_{x_{1}}-2,2} \\ a\sum_{i=1}^{L_{x_{1}}} \psi_{i,3}+\left( b_{3}\alpha+b_{2}\alpha+b_{1}\alpha+b_{2}\alpha\right)=E\psi_{L_{x_{1}}-1,2} \\ a\sum_{i=1}^{L_{x_{1}}} \psi_{i,3}+\left( b_{3}\alpha+b_{2}\alpha+b_{1}\alpha\right)=E\psi_{L_{x_{1}},2} \end{aligned} \right., \#\left( S11 \right) \end{aligned}$$

and we can obtain that

$$\begin{aligned} \left\{ \begin{aligned} \psi_{1,2}=\psi_{L_{x_{1}},2}=\tau+\left( b_{1}+b_{2}+b_{3} \right)\alpha/E=\beta\\ \psi_{2,2}=\psi_{L_{x_{1}}-1,2}=\tau+\left( b_{2}+b_{1}+b_{2}+b_{3} \right)\alpha/E=\eta\\ \psi_{3,2}=\psi_{4,2}=\ldots=\psi_{L_{x_{1}}-2,2}=\alpha/E\sum_{i=1}^{L_{x_{1}}} \psi_{i,3}=\tau\end{aligned} \right.. \#\left( S12 \right) \end{aligned}$$

By systematically solving the eigen-equations up to the $N$th sublattice (here $N=5$), we establish the relationship among different elements of the eigenstate. Starting from the $N$th system of equations, we sum both sides of all $N$ systems of equations. The chosen coefficients $\left\{ b_{1}=3, b_{2}=-2,b_{3}=0.5 \right\}$ satisfy the relation $b_{3}+b_{2}+b_{1}+b_{2}+b_{3}=0$, enabling simplification of the system. Through substitution and elimination of all $\psi$ terms, we derive an eighth-order polynomial equation in $E$:

$4E^{8}+32aE^{6}+\left( 96a^{2}-60a^{2}L_{x_{1}} \right)E^{4}+\left( 128a^{3}-240a^{3}L_{x_{1}}+168a^{3}L_{x_{1}}^{2} \right)E^{2}+ 64a^{4}+240a^{4}L_{x_{1}}+436a^{4}L_{x_{1}}^{2}-429a^{4}L_{x_{1}}^{3}=0$. $\left( S13 \right)$

The results are the eigenvalues of the 1^st^ EB mode, from which the corresponding eigenstate expressions can be derived. According to these analyses, substituting the trial wave function with undetermined coefficient $\gamma$ into the eigenequation, we obtain the fitting expressions of EB quasi-flat eigenstates of the 1D EB system in the following:

$\left| \psi_{R} \right\rangle\approx\frac{1}{\sqrt{L_{x_{1}}}}\left| 1,\ldots1 \right\rangle_{L_{x_{1}}}\otimes\left| 1,\gamma,\ldots,\gamma^{N-1} \right\rangle_{N}$, $\left( S14 \right)$

$\left\langle\psi_{L} \right|\approx\frac{1}{\sqrt{L_{x_{1}}}}\left\langle1,\ldots1 \right|_{L_{x_{1}}}\otimes\left\langle\gamma^{N-1},\gamma^{N-2},\ldots,1 \right|_{N}$, $\left( S15 \right)$

where $\left| 1,\ldots1 \right\rangle_{L_{x_{1}}}$ is column vector with $L_{x_{1}}$ elements. The coefficient $\gamma$ is $\gamma=\gamma_{1}{L_{x_{1}}}^{\theta_{1}}a^{-1/2}$ where the parameters $\gamma_{1}$ and $\theta_{1}$ are determined by couplings strengths $a_{l}$ and $b_{l}$. When $a={10}^{6}$ and $b_{1}=3,b_{2}=-2, b_{3}=0.5$, the parameters now taken as $\gamma_{1}\approx2.26,\theta_{1}\approx-0.66$. It should be noted that the above results lacks edge corrections, approximating the edge states with bulk distributions (e.g., in Eq. (S12), $\tau$ entirely replaces $\beta$ and $\eta$). For the spatial profiles of the sublattice, the numerical solutions are in good agreement with the results expressed by Eq. (S14) and Eq. (S15) as shown in Fig .S2.

**Figure S2.** The comparison between the results of 1^st^ EB state after numerical and the fitting results in 1D case. The blue line is the result of EB state distribution by the numerical calculation, and the orange line is the fitting result of EB state distribution with $\gamma_{1}\approx2.26,\theta_{1}\approx-0.66$.

In addition, we also plot the EB state and eigenvalues with different order $N$ as shown in Fig. S3. With the increase of $N$, the number of EB states also increase, remaining isolated from trivial states. Moreover, the spatial distribution of EB state almost unchanged and still exhibits a flat distribution localized on a single sublattice.


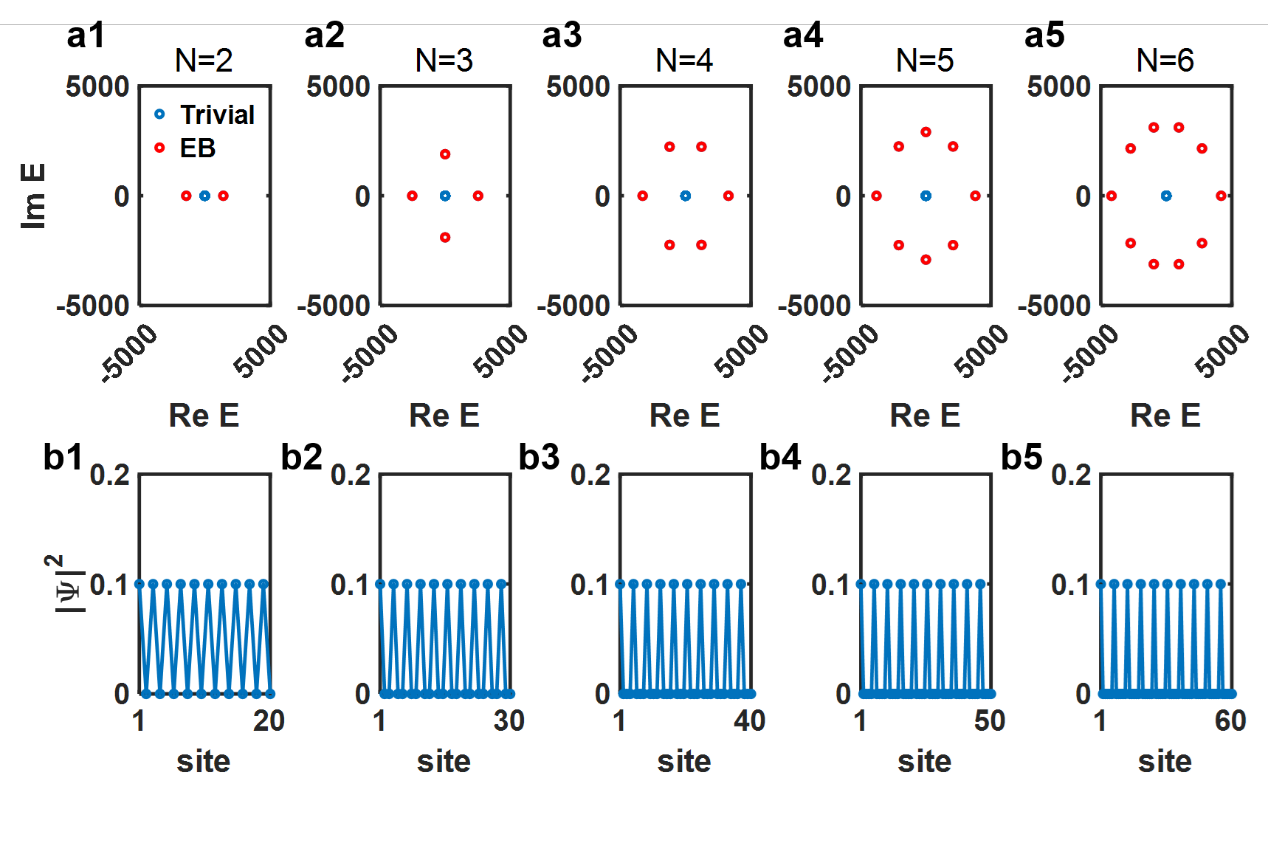


**Figure S3.** The eigenvalues and distributions of EB states with different orders.

**2.2 Two-dimensional case**

We now examine the 2D EB system Hamiltonian $P_{2D}$ in real space, as defined in Eq. (S4). Similar to the 1D case, we have performed eigenstate fitting for this 2D system:

$\left| \psi_{R} \right\rangle_{2D}=\left| \psi_{R} \right\rangle\otimes\left| 1,\ldots1 \right\rangle_{L_{x_{2}}}$, $\left( S16 \right)$

where $\left| \psi_{R} \right\rangle\approx\frac{1}{\sqrt{L_{x_{1}}}}\left| 1,\ldots1 \right\rangle_{L_{x_{1}}}\otimes\left| 1,\gamma,\ldots,\gamma^{N-1} \right\rangle_{N}$ is shown in Eq. (S14), and the $\left| 1,\ldots1 \right\rangle_{L_{x_{2}}}$ describes the states along $x_{2}$ direction. The numerically obtained eigenstates of the 2D EB system are shown in Fig. S4a, which have the excellent correspondence with the fitting results of Eq. (S16), see Fig. S4b.


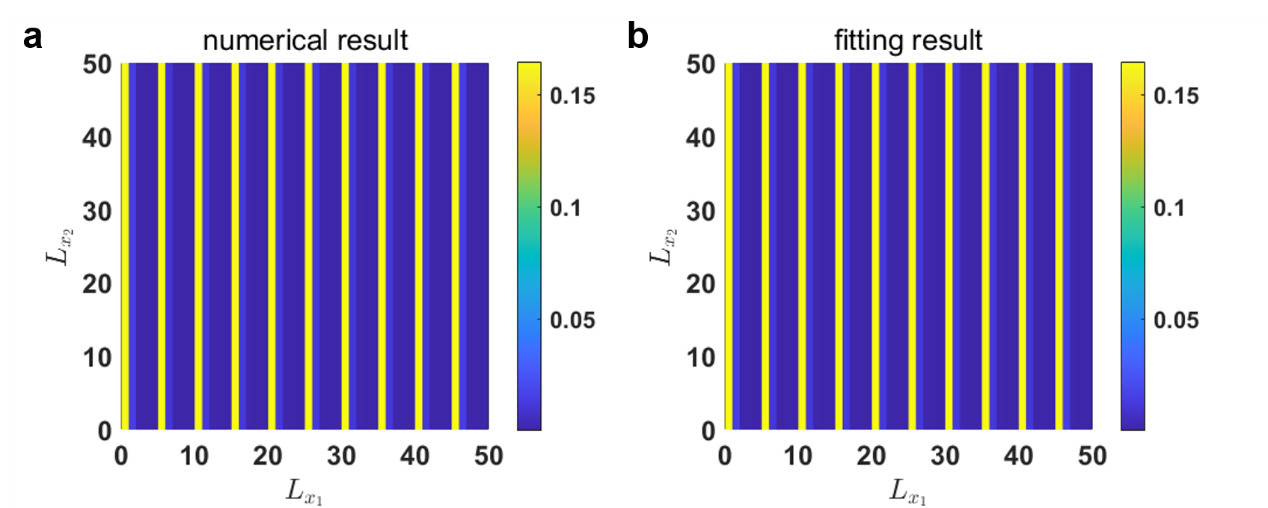


**Figure S4**. The comparison between the results of 1^st^ EB state after numerical and the fitting results in 2D EB system with $L_{x_{1}}=50,L_{x_{2}}=50.$

**Supplementary Information S3. The construction of the high dimensional EB** **systems.**

As evidenced by the preceding analyses in both 1D and 2D cases, the proposed method for constructing the EB system exhibits broad applicability, as it does not impose any inherent constraints on the dimensionality of the Hamiltonian $H_{0}$. In fact, for the $M$-dimensional EP system $H_{0}$ in momentum-space, we can construct the corresponding $M$-dimensional EB system $P_{\boldsymbol{k}}$ and the corresponding form $P$ in real space by following the same construction procedure presented above. Similarly, $P_{k}=\frac{1}{2}\left( \mathbb{I+}\frac{H_{0}}{E_{0}} \right)$ and $P=\sum_{\boldsymbol{k'}} \left| \boldsymbol{k'} \right\rangle\left\langle\boldsymbol{k'} \right|\otimes P_{\boldsymbol{k'}}$, where $\left| \boldsymbol{k'} \right\rangle=\frac{1}{\sqrt{L}}\sum_{\boldsymbol{k'}} e^{i\boldsymbol{x}\cdot\boldsymbol{k'}}\left| \boldsymbol{x} \right\rangle$ is basis vector in momentum-space, which is obtained by inverse Fourier transform of real space basis vector for unit cell $\left| \boldsymbol{x} \right\rangle$. Here,$L=\prod_{s=1}^{M} L_{x_{s}}$ is the scale of the whole $M$-dimensional system, where $L_{x_{s}}$ is the number of unit cells in $x_{s}$-direction of the system.$\boldsymbol{k}^{\boldsymbol{'}}\boldsymbol{=k+\Delta}$, where $\boldsymbol{k}=\left( k_{x_{1}},k_{x_{2}},\ldots k_{x_{M}} \right)$ represents the $M$-dimensional momentum and is chosen to take on discrete value from the first Brillouin zone. That momentum is related to the index $\boldsymbol{x}=\left( x_{1},x_{2},\ldots x_{M} \right)$ in $M$-dimensional real space by the Fourier transform. A small offset $\boldsymbol{\Delta}$ in the momentum space is chosen to avoid the singularity caused by the degenerate at the EP. In this way, the element of $P$ that represents the couplings from $\left( \boldsymbol{l}_{\mathbf{2}},\alpha_{j} \right)$ to $\left( \boldsymbol{l}_{\mathbf{1}},\alpha_{i} \right)$ in real space can be expressed as:

$P_{\left( \boldsymbol{l}_{\mathbf{1}},\alpha_{i} \right)\left( \boldsymbol{l}_{\mathbf{2}},\alpha_{j} \right)}=\sum_{k'} \left\langle\alpha_{i} \right|P_{k'}\left| \alpha_{j} \right\rangle e^{-i\boldsymbol{k}\boldsymbol{'\cdot}\left( \boldsymbol{l}_{\mathbf{1}}\mathbf{-}\boldsymbol{l}_{\mathbf{2}} \right)}$, $\left( S17 \right)$

where $\boldsymbol{l}_{\boldsymbol{1}}$ refers to the unit cell of the $M$-dimensional real space, whose location is marked by $\boldsymbol{l}_{\boldsymbol{1}}=[x_{1}(l_{1}),x_{2}(l_{1}),\ldots x_{M}(l_{1})]$, while $x_{1}(l_{1})$ represents the real space coordinate of the first dimension $x_{1}$ of $\boldsymbol{l}_{\boldsymbol{1}}$, and the others are similar. Besides each unit cell has $N$ sublattice sites and the $i$th sublattice is marked by $\alpha_{i}\left( 1\leq i\leq N \right)$. $\boldsymbol{l}_{\boldsymbol{2}}$ refers to the unit cell marked by $\boldsymbol{l}_{\boldsymbol{2}}=[x_{1}(l_{2}),x_{2}(l_{2}),\ldots x_{M}(l_{2})]$, where corresponding the $j$th sublattice of $N$ subcells is $\alpha_{j}\left( 1\leq j\leq N \right)$. According to the discussion above, this construction methods remain valid when employing the Fourier transform in a larger dimensional space, providing a universal framework for describing systems of arbitrary dimensions.

**Supplementary Information S4.** **Robustness of EB states for 1D and 2D system.**

For the 1D system $P_{1D}$ with $L_{x_{1}}=300$, when one end of the chain is fixed and the system is truncated, the eigenvalues of the resulting finite chain $P_{cut}=\mathcal{R}P_{1D}\mathcal{R}$ exhibit noticeable deviations from those of the original system $P$, where $\mathcal{R}$ is projection matrix, where the first $N\times x_{cut}$ diagonal elements are 1, and the remaining terms are zero. For example, when $x_{cut}=1$, $P_{cut}=\left( \begin{matrix} \boldsymbol{A}_{1} & \boldsymbol{0} \\ \boldsymbol{0} & \boldsymbol{0} \end{matrix} \right)_{NL_{x_{1}}}$ and when $x_{cut}=2$, $P_{cut}=\left( \begin{matrix} \boldsymbol{A}_{1} & \boldsymbol{A}_{2} & \boldsymbol{0} \\ \boldsymbol{A}_{2} & \boldsymbol{A}_{1} & \boldsymbol{0} \\ \boldsymbol{0} & \boldsymbol{0} & \boldsymbol{0} \end{matrix} \right)_{NL_{x_{1}}}$.

Similarly, as $x_{cut}$​ varies continuously, the size of our truncated system changes accordingly, leading to corresponding continuous variations in the eigenvalues of the system $E_{cut}$. Therefore, the eigenvalues of $P_{cut}$ evolves as the position of its truncation $x_{cut}$, and induces the EB spectral flow that we can observe the continuous change of the eigenvalues of the system.

In addition to analyzing the spectral flow, we investigate the robustness of the system against random coupling disorder. For the 1D EB $\boldsymbol{P}$, we introduce disorder by multiplying all coupling terms by a random factor $F_{ij}=1+r_{R}$, where $r_{R}$ is a random variable uniformly distributed over the interval $\left[ -\mu,\mu\right]$. Here, $\mu$ parameterizes the strength of the disorder. Then we consider the disorder system $\overline{P}$ with elements:

$\overline{P_{ij}}=P_{ij}*F_{ij}$ $\left( S18 \right)$

It is obvious that the $\overline{P}$ obtained each time is random, and the corresponding eigenvalues will also change differently. After repeating enough times, these eigenvalues will cover the possible disordered change results, showing the characteristics of 'eigenvalue cloud '. After considering several ranges of disorders as $\mu=0.01$, 0.02, 0.05 and 0.1 and repeating 40 times each value of disorder, we can obtain the resulting eigenvalues which form a spectral cloud in Fig. 2**b** of the main text.

Besides, the EB spectral flow of the 2D system under spatial truncation and the robustness of the system against random coupling disorder are also displayed in Fig. S5. It can be observed that these results are generally consistent with the 1D case presented in the main text, with the primary distinction being that only the 1^st^ EB state remains clearly resolvable in the 2D system.


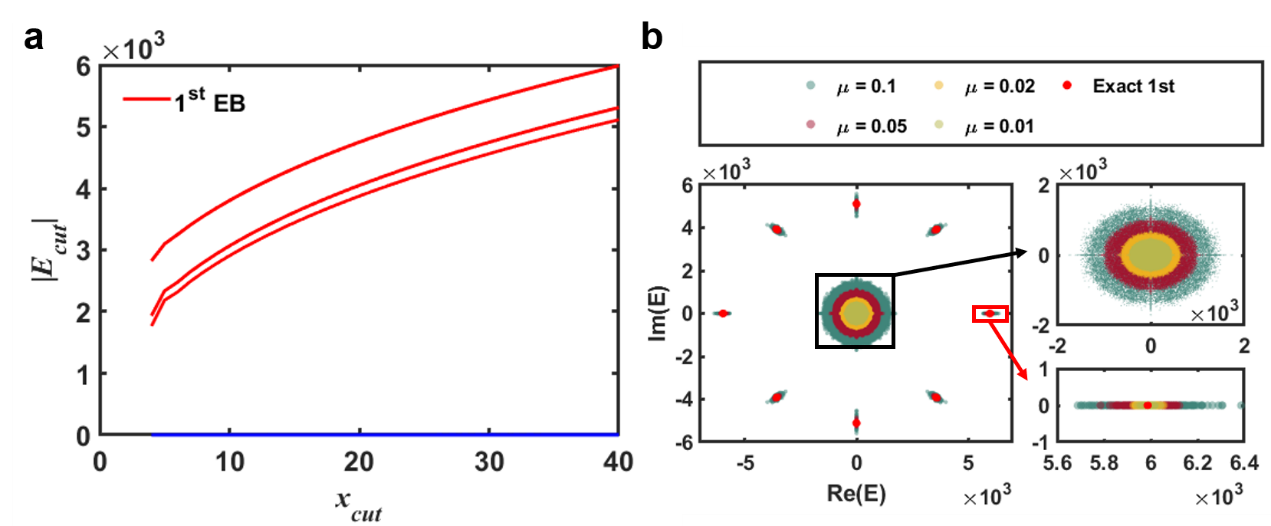


**Figure S5.** Robustness of EB states and their spectral flow. (a) The spectral flow of the absolute value of eigenvalues $\left| E_{cut} \right|$ for 2D system as the un-truncated region $\left[ x_{L},x_{R} \right]=\left[ 1,x_{cut} \right]$ is varied. (b) Distribution of the complex $E$ eigenvalues from 20 instances of different random disorders in the *P.* The parameters are $L_{x_{1}}=40$ and $L_{x_{2}}=10$ with disorders taken uniformly from $\left[ 1-\mu,1+\mu\right]$.

**Supplementary Information S5. Details of the fitting process under the perturbation.**

**5.1 One-dimensional case**

In this part, we provide the details of the fitting results. The detailed fitting parameters and corresponding results from the analysis are systematically summarized in Table I.

Table I. The fitting coefficients with 95% confidence bounds

|  | $\kappa_{2}\left( N \right)$ (small $\Gamma$) | $\kappa_{2}\left( N \right)$ (large $\Gamma$) | $\kappa_{3}\left( N \right)$ (small $a$) | $\kappa_{3}\left( N \right)$ (large $a$) | $\kappa_{4}\left( N \right)$ |
| --- | --- | --- | --- | --- | --- |
| $N=5$ | $0.9980\pm0.0002$ | $0.2162\pm0.012$ | $2.012\pm0.006$ | $0.8059\pm0.0008$ | $1.257\pm0.012$ |
| $N=6$ | $1.0041\pm0.0005$ | $0.1806\pm0.013$ | $2.506\pm0.003$ | $0.8378\pm0.0003$ | $0.7268\pm0.0039$ |
| $N=7$ | $0.9961\pm0.0005$ | $0.1547\pm0.013$ | $3.002\pm0.001$ | $0.8586\pm0.0001$ | $0.7364\pm0.0012$ |
| $N=8$ | $1.0379\pm0.0052$ | $0.1352\pm0.012$ | $3.501\pm0.002$ | $0.8757\pm0.0001$ | $0.8086\pm0.104$ |
|  | $\Delta E\propto\Gamma$ | $\Delta E\propto\Gamma^{1/N}$ | $\Delta E\propto a^{\left( N-1 \right)/2}$ | $\Delta E\propto a^{\left( N-1 \right)/N}$ | $\Delta E\propto L^{\theta_{1}\left( 1-N \right)+1}$ |

As the fitting procedure shown in Methods I, we first establish the relation between $\Gamma$ and $N$ with an empirical scaling law that $\Delta E\propto\Gamma^{\kappa_{2}\left( N \right)}$ for fixed $a$ and $L_{x_{1}}$ $\left( a={10}^{6},L_{x_{1}}=40 \right)$. At small $\Delta E$ regime, the obtained coefficients $\kappa_{2}\left( N \right)$ for $\Delta E$ versus $\Gamma$ is modeled as $\Delta E\propto\Gamma$. At large $\Delta E$ regime, the coefficients $\kappa_{2}\left( N \right)$ for $\Delta E$ versus $\Gamma$ is modeled as $\Delta E\sim\Gamma^{1/N}$. The detailed data are shown in Table I. The explicit relations between $\Delta E$ versus $\Gamma$ have been shown in Fig. S6.


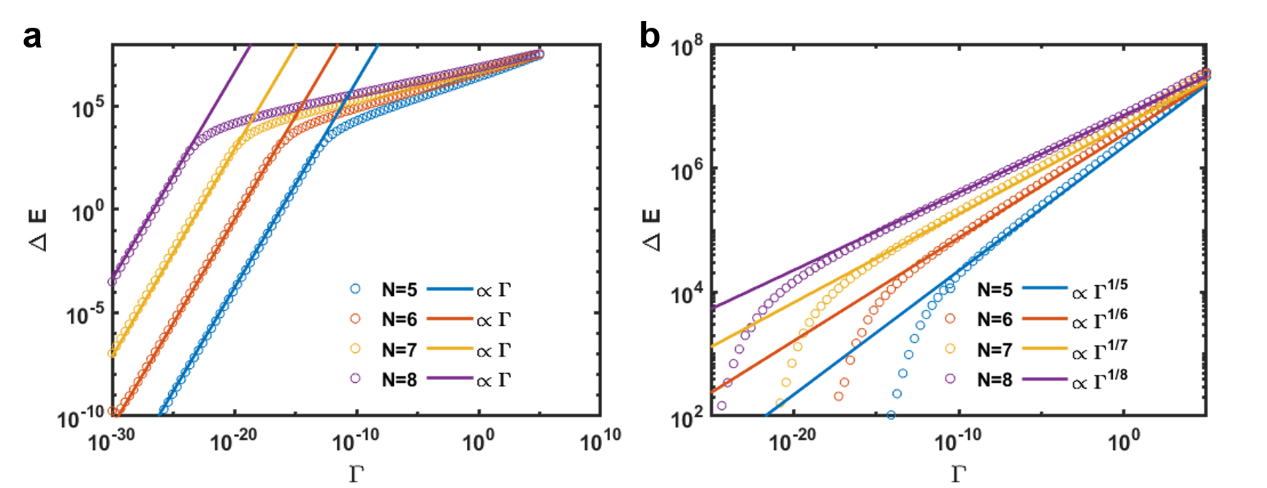

**Figure S6.** The relationship between $\Delta E$ and $\Gamma$ with various order $N$. (a) The relationship $\Delta E\propto\Gamma$ when $\Gamma$ is relatively small. (b) The relationship $\Delta E\propto\Gamma^{1/N}$ when $\Gamma$ is relatively large.

Then for fixed $\Gamma$ and $L$ $\left( \Gamma={10}^{-25},L_{x_{1}}=40 \right)$, the relationship between $\Delta E$ and $a$ is fitted using the functional form $\Delta E\propto a^{\kappa_{3}\left( N \right)}$. For different orders $N$, when $a$ is relatively small, the obtained coefficients of exponents $\kappa_{3}\left( N \right)$ for $\Delta E$ versus $a$ is modeled as $\Delta E\sim a^{\left( N-1 \right)/2}$ . And when $a$ is relatively large, the coefficients $\kappa_{3}\left( N \right)$ for $\Delta E$ versus $a$ is modeled as $\Delta E\propto a^{\left( N-1 \right)/N}$. The explicit relations between $\Delta E$ versus $a$ have been shown in Fig. S7.


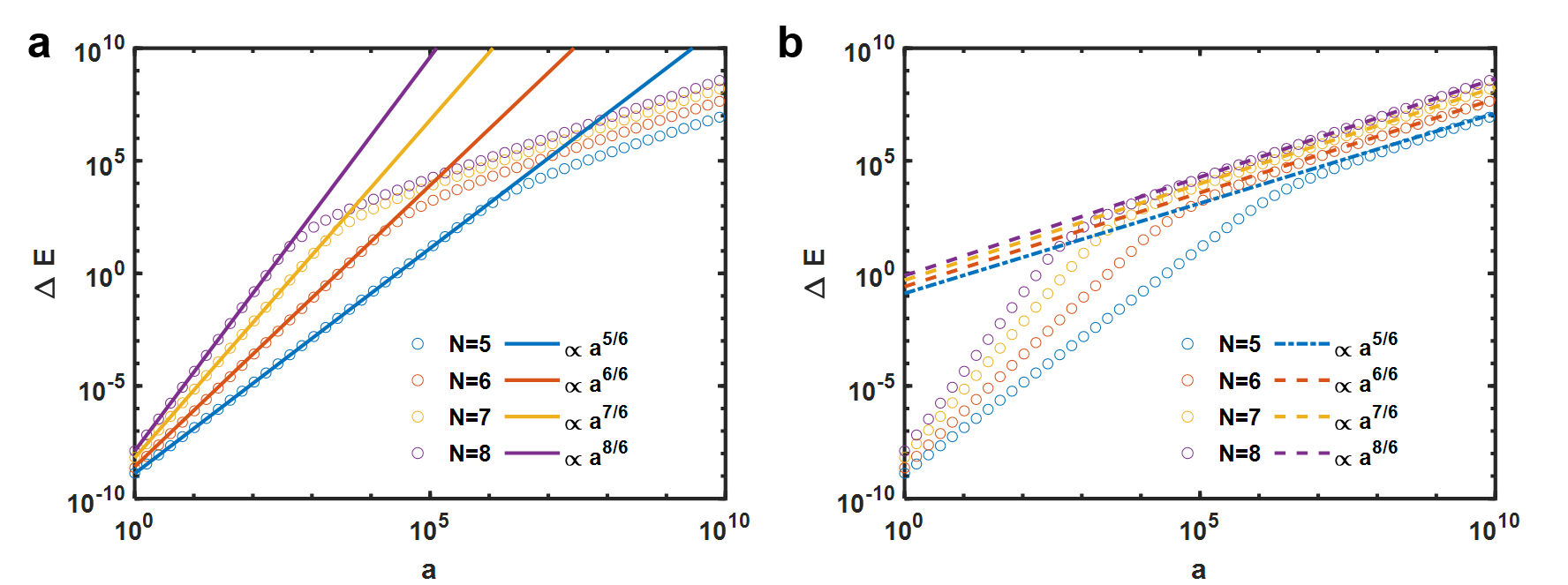


**Figure S7.** The relationship between $\Delta E$ and $a$ with various order $N$. (a) The relationship $\Delta E\propto a^{\left( N-1 \right)/2}$ when $a$ is relatively small. (b) The relationship $\Delta E\propto a^{\left( N-1 \right)/N}$ when $a$ is relatively large.

For fixed $\Gamma$ and $a$ $\left( a={10}^{6}, \Gamma={10}^{-25} \right)$, the size dependence $\Delta E\sim L_{x_{1}}$ reveals finite-size scaling. Similarly, for the relationship between $\Delta E$ and $L_{x_{1}}$ with fitted functional form $\Delta E\propto L^{\theta_{1}\left( N-1 \right)+1}$.

Then the comprehensive scaling relations can be obtained as:

$\Delta E\propto\left\{ \begin{matrix} \Gamma a^{\left( N-1 \right)/2}{L_{x_{1}}}^{\theta_{1}\left( 1-N \right)+1} & small \Delta E \\ \Gamma^{1/N}a^{\left( N-1 \right)/N}{L_{x_{1}}}^{\theta_{1}\left( 1-N \right)+1} & large \Delta E \end{matrix} \right.$ (S19)

In addition, we can also give a theoretical derivation to obtain the same results of the small $\Delta E$ part in Eq. S19 by considering the the first order effect using perturbation theory. For the unperturbed EB Hamiltonian, we can obtain the 1D EB eigenstates of Eq. S14 and Eq. S15 in Supplementary Information:

$$\begin{aligned} \left| \psi_{R} \right\rangle\approx\frac{1}{\sqrt{L_{x_{1}}}}\left| 1,\ldots1 \right\rangle_{L_{x_{1}}}\otimes\left| 1,\gamma,\ldots,\gamma^{N-1} \right\rangle_{N}\#(S20) \\ \left\langle\psi_{L} \right|\approx\frac{1}{\sqrt{L_{x_{1}}}}\left\langle1,\ldots1 \right|_{L_{x_{1}}}\otimes\left\langle\gamma^{N-1},\gamma^{N-2},\ldots,1 \right|_{N}\#(S21) \end{aligned}$$

The overlap between the left and right states is

$$\begin{aligned} \left\langle\psi_{L} | \psi_{R} \right\rangle=N\gamma^{N-1}=N{\gamma_{1}}^{N-1}{L_{x_{1}}}^{\theta_{1}\left( N-1 \right)}a^{-\left( N-1 \right)/2}\#(S22) \end{aligned}$$

with $\gamma=\gamma_{1}{L_{x_{1}}}^{\theta_{1}}a^{-1/2}$. We introduce a perturbation $\Delta P_{1D}$ shown in Fig. 3a in the main text to represent the non-Hermitian coupling between the specified sublattices. According to the first-order perturbation theory for non-Hermitian systems, the leading-order shift is given by:

$$\begin{aligned} \begin{aligned} \begin{aligned} \Delta E&\approx\frac{\left\langle\psi_{L} | \Delta P_{1D} | \psi_{R} \right\rangle}{\left\langle\psi_{L} | \psi_{R} \right\rangle}=\frac{\Gamma/{L_{x_{1}}}*L_{x_{1}}\left( L_{x_{1}}-1 \right)}{N{\gamma_{1}}^{N-1}{L_{x_{1}}}^{\theta_{1}\left( N-1 \right)}a^{-\left( N-1 \right)/2}}\# \\ &\approx\frac{1}{N{\gamma_{1}}^{N-1}}\Gamma a^{\left( N-1 \right)/2}{L_{x_{1}}}^{\theta_{1}\left( 1-N \right)+1}\#(S23) \\ &\propto\Gamma a^{\left( N-1 \right)/2}{L_{x_{1}}}^{\theta_{1}\left( 1-N \right)+1} \end{aligned}\# \end{aligned}\# \end{aligned}$$

These results demonstrate a close agreement between the numerical fit and the theoretical derivation, which not only validates the effectiveness of the theoretical framework in describing the results of perturbations, but also confirms the applicability of the numerical method.

And the results of our EB sensor can also be compared with SSH sensor:

$$\begin{aligned} \left| \Delta E_{SSH} \right|=\Gamma\frac{\left( r_{L}r_{R}-1 \right)\left( r_{L}^{L_{x_{1}}-1}+r_{R}^{L_{x_{1}}-1} \right)}{\left( r_{L}r_{R} \right)^{L_{x_{1}}}-1}\underset{\to}{L_{x_{1}}\gg1}\Gamma\frac{1}{6}e^{\log1.5*L_{x_{1}}}\#(S24) \end{aligned}$$

with $r_{L}=-1.5$, $r_{R}=-0.5$. When taking$L_{x_{1}}=10$ and $\Gamma={10}^{-15}$，$\left| \Delta E_{SSH} \right|\approx{10}^{-14}$, and the EB sensor reaches $\Delta E_{EB}\approx{10}^{-2}$ according to Eq. (S19), which display the improvement of 12 orders of magnitude.

**5.2 Two-dimensional case**

For the two-dimensional case, we follow the same fitting procedure and obtain analogous fitting results for the 2D system.

Table II. The fitting coefficients with 95% confidence bounds for 2D system.

|  | $\kappa_{2}\left( N \right)$ (small $\Gamma$) | $\kappa_{2}\left( N \right)$ (large $\Gamma$) | $\kappa_{3}\left( N \right)$ (small $a$) | $\kappa_{3}\left( N \right)$ (large $a$) | $\kappa_{4}\left( N \right)$ |
| --- | --- | --- | --- | --- | --- |
| $N=5$ | $0.9990\pm0.0010$ | $0.2043\pm0.031$ | $2.053\pm0.013$ | $0.8179\pm0.028$ | $1.205\pm0.112$ |
| $N=6$ | $0.9881\pm0.0042$ | $0.1729\pm0.027$ | $2.486\pm0.008$ | $0.8327\pm0.046$ | $0.7468\pm0.163$ |
| $N=7$ | $1.0521\pm0.0037$ | $0.1411\pm0.044$ | $3.024\pm0.005$ | $0.8593\pm0.031$ | $0.7064\pm0.247$ |
| $N=8$ | $1.0134\pm0.0002$ | $0.1291\pm0.052$ | $3.527\pm0.007$ | $0.8704\pm0.0017$ | $0.8386\pm0.054$ |
|  | $\Delta E\propto\Gamma$ | $\Delta E\propto\Gamma^{1/N}$ | $\Delta E\propto a^{\left( N-1 \right)/2}$ | $\Delta E\propto a^{\left( N-1 \right)/N}$ | $\Delta E\propto L^{\theta_{2}\left( 1-N \right)+1}$ |

The comprehensive scaling relations are:

$\Delta E\propto\left\{ \begin{matrix} \Gamma a^{\left( N-1 \right)/2}L^{\theta_{2}\left( 1-N \right)+1} & small \Delta E \\ \Gamma^{1/N}a^{\left( N-1 \right)/N}L^{\theta_{2}\left( 1-N \right)+1} & large \Delta E \end{matrix} \right.$ (S25)

**Supplementary Information S6. Calculation process of signal-to-noise ratio.**

We now analyze the quantum noise characteristics of the EB model and evaluate the corresponding signal-to-noise ratio (SNR). The full quantum calculations are based on Heisenberg–Langevin equations and are solved numerically using the Runge-Kutta method with adaptive time steps, while the noise is modeled as a Wiener process. Using input–output theory, a signal is extracted from the system for which the SNR is derived:

$SNR=\frac{\left| {\partial\omega}/{\partial\Delta\omega} \right|}{\sigma_{\omega}}$, (S26)

where $\left| {\partial\omega}/{\partial\Delta\omega} \right|$ is the susceptibility and $\sigma_{\omega}$ is he frequency uncertainty.

For each simulation run, it initiates with a warm-up, subsequently followed by data sampling for a fixed time interval $t$ with an independent noise realization. Numerical results on the variation of SNRs as a function of the coupling strength $a$ with structural disorder and background noise have been shown in Fig. S8. The strengths of structural disorder is considered as μ=0.05, with the background noise in is modeled as Gaussian white noise with a mean values of zero and the standard deviation $D=0.5$.

**Figure S8.** Numerical results on the variation of SNRs as a function of the coupling strength $a$ with structural disorder and background noise.

While the conclusion reached is that our sensor does not have an advantage in terms of quantum-limited SNR, the implementation of the EB model on our circuit platform provides an alternative approach for noise suppression, thereby enabling SNR enhancement. Details of analysis of SNR in the EB circuit sensor are provided in S9 of Supplementary Information.

**Supplementary Information S7.** **Printed Circuit Board (PCB) fabrications and measured results.**

To experimentally realize our EB system in circuit, we fabricated a printed circuit board (PCB). The complete implementation consists of four modular PCB units, corresponding to the theoretical parameter $L_{x_{2}}=4$ in our circuit. Each PCB unit contains $L_{x_{1}}^{2}=16$ submodules, where each submodule represents either intra-cell or inter-cell coupling in the theoretical lattice model. Due to the presence of long-range couplings in our model, where non-reciprocal intercell coupling exists between all unit cells, the $L_{x_{1}}\times L_{x_{1}}$​ submodules are necessary to implement these couplings.

The detailed PCB layout is illustrated in the accompanying figure, where Figure S9**a** corresponds to intracell coupling. Figure S9**b**, **c** and **d** represent different intercell coupling parts, respectively.

**Figure S9**. The detailed PCB structure diagram of the circuit we designed. A buffer (BUF634A) is employed to construct a voltage follower, ensuring unidirectional current flow and thereby realizing non-reciprocal coupling.

We fabricated the circuit structure shown in Fig. 4**a** of the main text on PCB and experimentally characterized its electrical properties. As shown in Figs. S10**a** and S10**b**, the measured eigenvalues and eigenstates of the circuit system demonstrate excellent agreement with the theoretical lattice model predictions. Furthermore, Figure S10**c** presents the eigenvalue variations under different values of resistor $R$, which also show strong consistency with the theoretically derived results.

**Figure S10**. The measured results of the designed circuit system. (a) and (b), no perturbation resistor *R* has been added. (a) The measured eigenvalues of the circuit, where the eigenvalues of 1^st^ EB modes depicted in red and other EB modes with trivial modes depicted in green. (b) The measured eigenstates of the circuit which distribute flat on the 2D spatial positions. (c) The measured eigenvalues of the circuit system with different values of resistor $R$ as perturbation.

**Supplementary** **Information S8. Robustness of EB circuit.**

The inherent robustness of EB states confers corresponding stability to sensing measurements utilizing these states. In Fig. S11, we show the eigenvalues of EB states with different fluctuations in circuit simulations. Even regarding the impact of component fluctuations, the eigenvalues of EB states still isolated from the those of trivial states.

**Figure S11**. The eigenvalues of EB states with different fluctuations in circuit simulations.

In addition, we introduced 20% disorder to all circuit components and performed 40 independent circuit simulations. As shown in Fig. S12, while the disorder significantly perturbs the eigenvalues of the systems, the frequency shifts of impedance peaks induced by the EB states still exhibit a sensitivity similar to case without disorder.


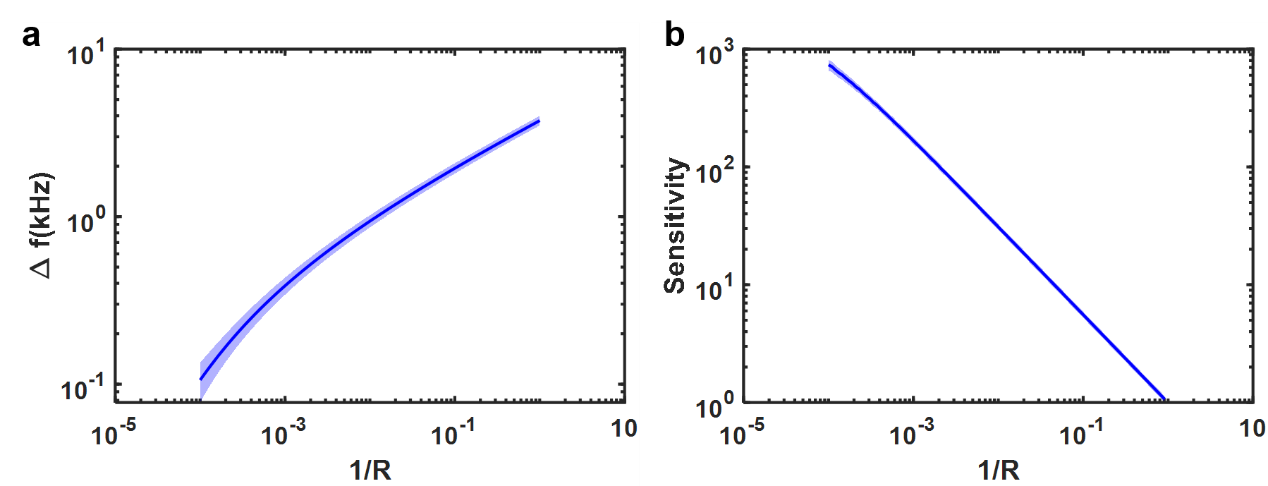


**Figure S12**. Robustness of the circuit. (a) The simulate results of the frequency shift $\Delta f$ with 20% disorder. The dark blue line is the mean result, and the blue shading is standard deviation. (b) The simulate results of the sensitivity with 20% disorder.

**Supplementary Information S9. Noise analysis of impedance spectra of designed circuits.**

In this part, we give the noise analysis of EB sensor circuits. The influence of noises on the designed sensors imposes the fundamental bound on the sensitivity. In the experiment, there are two primary noise sources in the measurement of frequency: (1) The parasitic errors of coupling devices in sensing systems, such as equivalent series resistance of capacitor. (2) Thermal noise.

For the parasitic errors, we note that the equivalent series resistance (ESR) and equivalent series inductance (ESL) of capacitor will affect the frequency characteristics of the capacitor. For instance, the idealized impedance term $i\omega C$ should be modified to $i\omega C+R_{ESR}+1/{i\omega L_{ESL}}$​. Therefore, we choose the capacitors with High Q and low ESR in high frequency range and consider its ESR and ESL in practice. Besides, the loss of inductor also significantly influences the impedance peak for resonance frequency, so we choose the inductors with appropriate series resistance. In addition, in order to ensure the accurate experimental implementation of our theoretically designed EB sensor circuit, the uncertainty of the components must be minimized so we have obtained the capacitors and inductors with 1% error tolerance from the capacitors with 10% error tolerance.

For the thermal noise, it mainly comes from resistors. For our circuit, the ESR of capacitor is approximately in the order of 10mΩ, and the series resistance of the inductor is near 100mΩ. Besides, the thermal noise in active devices buffers is also considered. And because of the noise gain of buffer is 1, the thermal noise is effectively suppressed.

Here, Figure S13a shows the measured SNR of the circuit system and the corresponding influence of noise on the impedance spectrum are displayed in Fig. S13b.


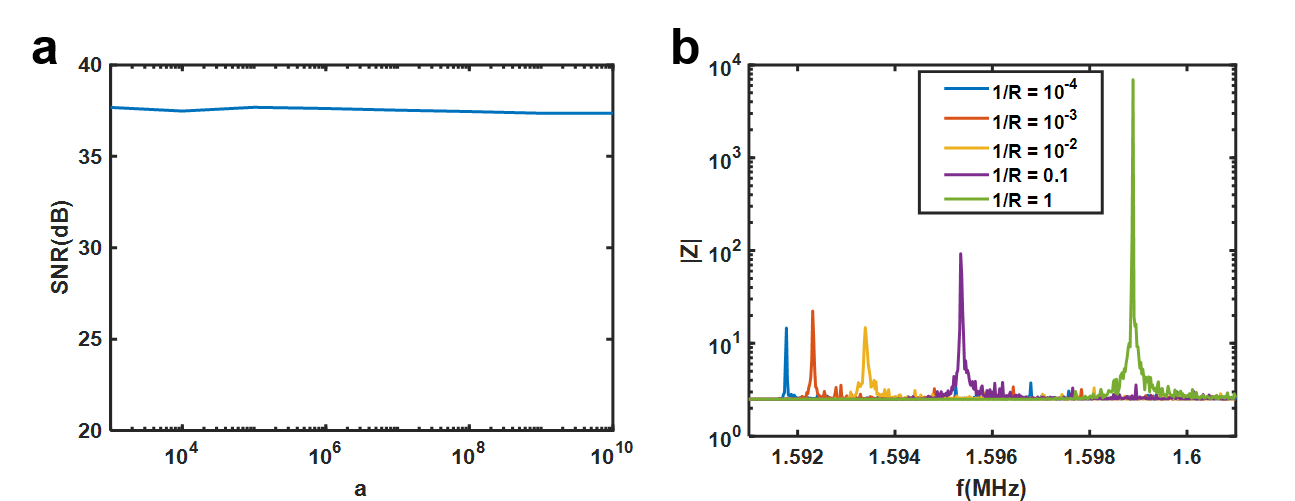


**Figure S13**. (a) The Signal-to-Noise Ratio (SNR) of the circuit system. (b) The influence of noise on the impedance spectrum.

**Supplementary Information S10.** **Detailed design methods and properties of the magnetic field measurement experiment.**

In general, the magnetic field can have an impact on a region, and therefore the signals on the region can be accumulated to be detected. For magnetic field generation, we employ a Helmholtz coil configuration, which consists of a pair of parallel, co-axial circular coils connected in series, with identical current magnitudes and directions. When the separation distance between the two coils equals their radius $r$, the system is referred to as a Helmholtz coil. This configuration generates a highly uniform magnetic field near the midpoint of the common axis. The magnetic flux density $B$ at any point along the axis is given by:

$$\begin{aligned} B=\frac{1}{2}\mu_{0}NIr^{2}\left\{ \left[ r^{2}+\left( \frac{r}{2}+x \right)^{2} \right]^{-\frac{3}{2}}+\left[ r^{2}+\left( \frac{r}{2}-x \right)^{2} \right]^{-\frac{3}{2}} \right\}\#\left( S27 \right) \end{aligned}$$

where $\mu_{0}$ denotes the vacuum permeability, $N$ is the number of coil turns, $I$ represents the current flowing through the coils, and $x$ is the axial coordinate with the origin $x=0$ set at the midpoint between the two co-axial coils. The magnetic flux density at the center $x=0$ is given by: $B_{0}=\left( \frac{4}{5} \right)^{\frac{3}{2}}\frac{\mu_{0}NI}{r}$. For our specific coil configuration ($N=183, r=0.15m$), this yields $B_{0}\approx1.1\times{10}^{-3}I$.

In addition to the magnetic field generation system, another key component is the magnetoresistive sensor, which converts magnetic field quantities into electrical signals. The high sensitivity of our designed circuit enables precise measurement of magnetic flux density. Here, we employed the HMC1001 magnetic sensor, configured as a four-element Wheatstone bridge with nickel-iron (Permalloy) thin-film resistors. When exposed to an external magnetic field, the bridge resistance changes due to the magnetoresistive effect. Our EB sensor circuit detects these resistance variations, with the output response to magnetic field strength given by:

$R\approx947.38+32.25\times B$. (S28)

Consequently, changes in the magnetic field strength can be amplified and converted into corresponding the frequency shifts in the circuit. In addition, in order to eliminate the influence of the geomagnetic field, we compensate by biasing the current to generate a reverse magnetic field.

For the circuit experiments, we employed the following instrumentation: A DC power supply (UNI-T UTP1306S) provided 15 V DC voltage to the buffer circuit; An arbitrary/function waveform generator (RIGOL DG1022Z) generated the required AC voltage signal; A digital storage oscilloscope (Agilent Technologies Infinii Vision DSO7104B) simultaneously monitored the voltage signals. Besides, to minimize measurement errors, we additionally used a WK6500B impedance analyzer to obtain frequency spectra of impedance, featuring: 0.05% basic accuracy for capacitance, inductance, and impedance measurements; ±0.0005 dissipation factor precision ±0.05 quality factor accuracy.
